# Hepatic NOD2 promotes hepatocarcinogenesis via a RIP2-mediated proinflammatory response and a novel nuclear autophagy-mediated DNA damage mechanism

**DOI:** 10.1186/s13045-020-01028-4

**Published:** 2021-01-07

**Authors:** Yi Zhou, Liang Hu, Wenqing Tang, Dongping Li, Lijie Ma, Hongchun Liu, Shuncai Zhang, Xiaojie Zhang, Ling Dong, Xizhong Shen, She Chen, Ruyi Xue, Si Zhang

**Affiliations:** 1 Department of Biochemistry and Molecular Biology, NHC Key Laboratory of Glycoconjugates Research, School of Basic Medical Sciences, Fudan University, Shanghai, 200032 China; 2 Department of Gastroenterology and Hepatology, Shanghai Institute of Liver Diseases, Zhongshan Hospital, Fudan University, Shanghai, 200032 China; 3 Department of Cardiology, the First Affiliated Hospital of Zhengzhou University, Cardiovascular Institute of Zhengzhou University, Zhengzhou, China; 4 Department of General Surgery, Zhongshan Hospital (South), Shanghai Public Health Clinical Center, Fudan University, Shanghai, China; 5 Department of Rheumatology, Qilu Hospital of Shandong University, Jinan, Shandong China

**Keywords:** Hepatocellular carcinoma, NOD2, Lamin A, C, RIP2, DNA damage, Nuclear autophagy

## Abstract

**Background:**

Key hepatic molecules linking gut dysbiosis and hepatocarcinogenesis remain largely unknown. Gut-derived gut microbiota contains pathogen-associated molecular patterns (PAMPs) that may circulate into the liver and, consequently, be recognized by hepatic pattern recognition receptors (PRRs). NOD2, a general intracellular PRR, recognizes muramyl dipeptide (MDP), present in both gram (+) and gram (−) bacteria. Here, we investigated the role of NOD2 as a molecular sensor translating gut dysbiosis signaling into hepatocarcinogenesis.

**Methods:**

NOD2 expression was measured in clinical hepatocellular carcinoma (HCC) samples using qPCR (80 pairs), western blotting (30 pairs) and immunostaining (141 pairs). The role of NOD2 in hepatocarcinogenesis was examined in the hepatocyte-specific *Nod2*-knockout (*Nod2*^*△hep*^), *Rip2*-knockout (*Rip2*^*△hep*^), *Lamin A/C*-knockout (*Lamn*^*△hep*^) and *Rip2*/*Lamin A/C* double-knockout (*Rip2/Lamn*^*△hep*^) mice models of diethylnitrosamine (DEN)/CCl_4_-induced HCC.

**Results:**

NOD2 was upregulated and activated in HCC samples, and high NOD2 expression correlated with poor prognosis in HCC patients. Hepatic NOD2 deletion in vivo decreased DEN/CCl_4_-induced HCC by reducing the inflammatory response, DNA damage and genomic instability. NOD2 activation increased liver inflammation via RIP2-dependent activation of the MAPK, NF-κB and STAT3 pathways. Notably, a novel RIP2-independent mechanism was discovered, whereby NOD2 activation induces the nuclear autophagy pathway. We showed that NOD2 undergoes nuclear transport and directly binds to a component of nuclear laminae, lamin A/C, to promote its protein degradation, leading to impaired DNA damage repair and increased genomic instability.

**Conclusions:**

We reveal a novel bridge, bacterial sensor NOD2, linking gut-derived microbial metabolites to hepatocarcinogenesis via induction of the inflammatory response and nuclear autophagy. Thus, we propose hepatic NOD2 as a promising therapeutic target against HCC.

## Introduction

Liver cancer was the fourth leading cause of cancer-related death worldwide in 2018 [1]. Hepatocellular carcinoma (HCC) constitutes 75–85% of all liver cancers and is closely associated with chronic liver injury, which leads to repetitive cell death and regeneration, as well as chronic liver inflammation and fibrosis, which causes DNA damage in hepatocytes. In response to DNA damage, the hepatocytes activate the DNA damage response (DDR) signal to repair DNA damage [2]. Disruption of the damage repair pathways may cause DNA mutations and transformation of normal cells into tumor cells [2], leading to genomic instability, and eventually, hepatocarcinogenesis.

Due to its close anatomical connection to the intestine via the portal vein, the liver is the first organ exposed to the gut microbiota and their metabolites, which contain pathogen-associated molecular patterns (PAMPs). There is increasing evidence showing that dysbiosis in the gut-liver axis may play an important role in hepatocarcinogenesis [3–5]. For instance, in HCC patients, and diethylnitrosamine (DEN)-induced HCC models, alteration of the gut microbiota, increases in intestinal permeability and translocation of bacterial metabolites from the gut to the liver have been reported [3–5]. Consequently, the gut-derived PAMPs may trigger persistent inflammation and hepatocellular injury in the liver, promoting HCC development. Furthermore, bacteria clearance using antibiotics has been shown to reduce HCC development in the DEN/CCl_4_-induced HCC model [4], suggesting that gut-derived PAMPs may participate in hepatocarcinogenesis. However, the mediators whereby the gut microbiota and their metabolites modulate liver tumorigenesis are still not well characterized.

We and others have previously shown that nucleotide-binding oligomerization domain 2 (NOD2), a member of pattern recognition receptors (PRRs), propagates the inflammatory response via RIP2 signaling in the circulatory system during bacterial invasion [6, 7]. Once gut-derived PAMPs translocate to the liver via circulation, the liver senses them mainly through the PRRs expressed on the hepatocytes [8]. NOD2 is a well-characterized intracellular PRR of the NOD-like receptor (NLR) family, consisting of a C-terminal leucine-rich repeat (LRR), a central nucleotide-binding (NATCH) domain and two N-terminal caspase activation and recruitment (CARD) domains [9]. Unlike Toll-like receptor 4 (TLR4), which recognizes the LPS found mainly in gram (−) bacteria [10], NOD2 senses muramyl dipeptide (MDP), which is present in both gram (−) and gram (+) bacteria, making it a broader sensor of bacterial invasion compared to other PRRs [11]. Therefore, certain genetic variants of *Nod2* gene are associated with an increased susceptibility to digestive disease such as Crohn’s disease [12, 13]. NOD2 has also been implicated in the liver inflammatory diseases, as evidenced by its role in the promotion of experimental cholestatic liver fibrosis and hepatitis through inflammatory cytokines production [14–16]. However, the role of hepatic NOD2 in HCC remains elusive.

In this study, we further reported that NOD2 is an important hepatic sensor of gut microbial metabolites that promotes hepatocarcinogenesis. We demonstrate that NOD2 promotes hepatocarcinogenesis through two different mechanisms, including a previously unrecognized role, whereby NOD2 initiates a nuclear autophagy pathway, leading to increased DNA damage and genomic instability.

## Methods

### Patient recruitment and HCC specimens

HCC specimens and paired adjacent non-tumor liver tissues were collected from 141 patients (Zhongshan Hospital HCC1, i.e., ZHH1 cohort, 2005–2010) receiving surgical resection at Zhongshan Hospital, Fudan University. Paired tumor and adjacent liver tissues were collected from 80 patients (ZHH2 cohort, 2012–2015) for mRNA analysis, 30 of which were randomly selected for western blot and immunohistochemistry (IHC) analyses. HCC specimens and normal liver tissues were collected from patients receiving surgical resection or liver transplantation at Zhongshan Hospital, Fudan University (*n* = 10). Postoperative follow-ups were performed as previously reported [17]. Overall survival (OS) was defined as the interval between the surgery and death or contact failure or the last investigation point [17]. Time to recurrence (TTR) is defined as the interval between surgery and the diagnosis of recurrence (intrahepatic recurrence or extrahepatic metastasis) [17]. This project was performed in accordance with the Declaration of Helsinki and approved by the Ethics Committee of the Zhongshan Hospital, Fudan University. Informed consents were obtained from all patients.

### Reagents, antibodies and cell line

Reagents used in this study include diethylnitrosamine (DEN, Sigma-Aldrich, St Louis, MO, USA), muramyl dipeptide (MDP, Invitrogen, San Diego, CA, USA), 3-methyladenine (3-MA, Selleck Chemicals, Shanghai, China), MG-132 (MedChemExpress, NJ, USA), Lyso-Tracker Red (Beyotime, Shanghai, China) and microsocial nuclease (New England Biolabs, Ipswich, MA, USA). The primary antibodies and dilutions used are listed in Additional file 1: Table S1. Biotinylated secondary antibodies were purchased from Santa Cruz; TRITC, Alexa Fluor 488 or Alexa Fluor 647-conjugated secondary antibodies were purchased from Jackson ImmunoResearch (West Grove, PA, USA). HEK293T cell line was originally purchased from the American Type Culture Collection (ATCC) and cultured in DMEM supplemented with 10% FBS, 100 U/ml penicillin and 100 μg/ml streptomycin in an incubator with 5% CO_2_ at 37 °C.

### Tissue microarrays (TMAs) and TCGA analysis

TMAs of the ZHH1 cohort were constructed as described previously [17]. To quantify protein expression levels based on TMAs, a scoring system was used based on the IHC staining signal intensity (0, negative; 1, weak; 2, moderate; 3, strong) and the percentage of positively stained cells (0, 0%; 1, 1–25%; 2, 26–50%; 3, 51–75%; 4, 76–100%) [17]. Composite expression scores were calculated by multiplying the scores of staining intensity and positive cell percentage and classified as negative (score 0), weak (scores 1–4), moderate (scores 5–8) and strong (scores 9–12) [17]. The IHC staining scores were independently evaluated by two pathologists in a double-blind manner. Receiver operating characteristic (ROC) curve analysis showed that the optimal cutoff value of NOD2 was 8, with the maximum area under the curve (AUC) of 0.691. Thus, NOD2 IHC score ≥ 8 was considered as high NOD2 expression, whereas score < 8 was defined as low.

RNA-Seq data of 371 HCC and 50 normal liver tissues were obtained from The Cancer Genome Atlas (TCGA) database (http://cancergenome.nih.gov) for analysis of NOD2 mRNA expression. OS was determined using Kaplan–Meier survival analysis; Gene Set Enrichment Analysis (GSEA) was performed using GSEA software (http://software.broadinstitute.org/gsea/downloads.jsp). For assessing the association of NOD2 expression with genetic instability in TCGA HCC cohort, 104 patients with TP53 mutation and 255 patients without TP53 mutations were retrieved for analysis.

### Animals and liver tumorigenesis

Mice with homozygous conditional-null alleles of *Nod2* (*Nod2*^*f/f*^) were generated with the help of the Model Animal Research Center, Nanjing University (Nanjing, China). Briefly, single-guide RNA (sgRNA), Cas9 mRNA and the donor vector targeting the exon 3 and 4 sites were microinjected into mouse zygotes to create a Nod2-flox allele (*Nod2*^*f/f*^) with two LoxP sites flanking exons 3 and 4. *Nod2*^*f/f*^ mice were bred to Albumin-Cre mice (Jackson Laboratory, Bar Harbor, ME; 003574) to generate hepatocyte-specific *Nod2*-knockout mice (*Nod2*^*△hep*^). Wild-type (WT) mice were purchased from Jackson Laboratory. *Rip2*^*f/f*^ (T005428) and *Lmna*^*f/f*^ mice (T007824) were purchased from GemPharmatech (Nanjing, China) and bred to Albumin-Cre mice to generate *Rip2*^*△hep*^, *Lmna*^*△hep*^ and *Lmna/Rip2*^*△hep*^ mice. Primers used for genotyping are listed in Additional file 1: Table S2. All animals are in the C57BL/6 background. All mice were provided with food and water ad libitum and maintained in a 12-h light–dark cycle under standard conditions at the Fudan University (Shanghai, China).

For chemically induced HCC, male mice aged 14–16 days were given a single intraperitoneal (i.p.) injection of DEN (25 mg/kg) and 8 subsequent biweekly injections of CCl_4_ (1.2 ml/kg, 1:4 diluted with olive oil, i.p., starting 4 weeks after DEN). Mice were killed at the indicated time points after DEN injection.

All experiments preformed were approved by the Research Ethics Committee of Zhongshan Hospital and conducted according to the institutional guidelines.

### Statistical analysis

Data were analyzed with SPSS 20.0 (SPSS Inc., Chicago, IL, USA). Unpaired Student’s *t* test or the Mann–Whitney U test was used for comparison between two groups. Paired *t* test was used to compare paired data. Analysis of variance (ANOVA) was used to compare 3 or more groups. Ordinary one-way or two-way ANOVA with a Sidak test was used to make multiple comparisons between different groups. Chi-square test was used for comparing categorical variables. Cox proportional hazard model was used for univariate and multivariate analyses. ROC curve analysis was used to determine the optimal cutoff value of the parameters by SPSS. Survival was analyzed by the Kaplan–Meier (log-rank) test. Pearson correlation analysis was used to assess the association between the expression of NOD2 and lamin A/C or p-RIP2. All data are expressed as mean ± standard deviation (SD). Difference between groups is considered statistically significant if *P* < 0.05.

A complete description of the methods, including histological analyses, isolation and culture of primary mouse hepatocytes, cell transfection and infection, reactive oxygen species (ROS) detection, ALT and AST analyses, quantitative polymerase chain reaction, western blot analysis, TaqMan Copy Number Analysis, MDP Concentration measurement, co-immunoprecipitation (co-IP) and mass spectrometry (MS) analysis, confocal microscopy, transmission electron microscope (TEM) and live-cell imaging, GST pull-down assay, subcellular fractionation, comet assay and non-homologous end joining (NHEJ) assay, is available in Additional file 2: Online materials and methods.

## Results

### Increased NOD2 expression is positively associated with aggressive phenotypes and poor prognosis of HCC

**Fig. 1:**
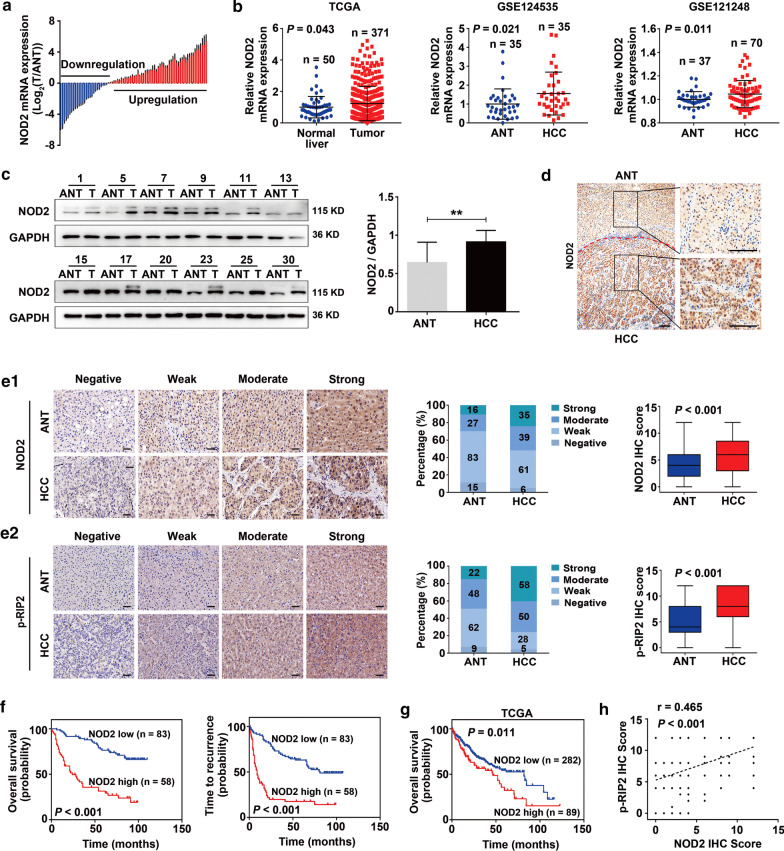
Increased NOD2 expression correlates with poor prognosis of HCC.** a** Relative NOD2 mRNA expression in 80 paired HCC (T) and adjacent non-tumor tissues (ANT) from Zhongshan Hospital HCC2 (ZHH2). **b** NOD2 mRNA expression in HCC cohorts from TCGA and GEO (GSE124535 and GSE121248) databases. **c** Representative expression pattern (left) and relative intensity (right) of NOD2 protein in 30 paired HCC and ANT tissues from ZHH2. **d** Representative immunohistochemistry images of NOD2 in HCC and ANT tissues. **e** Immunohistochemistry staining of NOD2 (**e1**) and p-RIP2 (**e2**) in 141 paired HCC and ANT tissues from ZHH1 (*n* = 141). **f** Kaplan–Meier analysis for OS and TTR according to NOD2 expression in HCC patients from ZHH1. **g** Kaplan–Meier analysis for OS in TCGA HCC cohorts. **h** Positive correlation between NOD2 scores and p-RIP2 scores in HCC samples from ZHH1, *n* = 141; Pearson correlation analysis. Data were shown as mean ± SD, and significance was determined using paired (**c**,** e**), and unpaired Student’s t test (**b**). ** *P* < 0.01. Scale bar, 50 μm

To determine the role of NOD2 in HCC development, we first examined its expression pattern in HCC samples (ZHH2, *n* = 80) using qPCR. Compared to the matched adjacent non-tumor (ANT) liver tissues, NOD2 expression was increased in 67.5% (54/80) of HCC samples (Fig. 1a), which was confirmed in the analysis of an HCC cohort using TCGA database and in the analysis of two cohorts (GSE124535, GSE121248) from Gene Expression Omnibus (GEO, https://www.ncbi.nlm.nih.gov/gds) datasets (Fig. 1b). Western blots of 30 randomly selected HCC samples from the ZHH2 cohort demonstrated increased NOD2 protein in HCC (Fig. 1c). In addition, IHC analyses of TMAs from the ZHH1 cohort (*n* = 141) indicated that HCCs have increased NOD2 protein expression compared to ANT tissues (Fig. 1d, e1), with an average score of 6.11 ± 3.69 for HCC and 4.19 ± 2.88 for ANT tissues (*P* < 0.001). Notably, phosphorylated RIP2 (p-RIP2), a downstream indicator of NOD2 activation, was also higher in HCCs than in ANT tissues (average score 7.97 ± 3.49 vs. 5.53 ± 3.25, *P* < 0.001; Fig. 1e2). Furthermore, NOD2 level was positively correlated with p-RIP2 in HCC (Fig. 1h). Consistently, both western blot and IHC analyses demonstrated that NOD2 levels increase gradually during the progression of DEN/CCl_4_-induced HCC, which is also accompanied by an increase in RIP2 phosphorylation (Additional file 3: Fig. S1), indicating NOD2 activation in HCC.

To determine the clinical significance of NOD2 in HCC, we examined the association between NOD2 expression and clinicopathological parameters in the ZHH1 cohort (*n* = 141). HCC tumors were divided into NOD2-high and NOD2-low group based on the cutoff value of their IHC scores (Additional file 3: Fig. S2). We found that the NOD2 expression level positively correlated with tumor aggressive phenotypes, including severe cirrhosis (*P* = 0.012), frequent vascular invasion (*P* = 0.007), multiple tumor number (*P* = 0.003), large tumor size (*P* = 0.032), high alpha fetoprotein (AFP, *P* = 0.014), advanced Edmondson grade (*P* = 0.002) and BCLC stage (*P* = 0.029) (Additional file 1: Table S3). Kaplan–Meier plot showed that NOD2-high patients have a significantly shorter median OS (33.72 vs. 74.17 months, *P* < 0.001) and TTR (18.84 vs. 58.20 months, *P* < 0.001) than NOD2-low patients (Fig. 1f). The shorter median OS in NOD2-high patients was confirmed in the analysis of the TCGA cohort (46.57 vs. 81.87 months, *P* = 0.011, Fig. 1g, *n* = 371). Univariate and multivariate analysis demonstrated that, apart from vascular invasion and tumor number, NOD2 expression remains as an independent prognostic factor of both OS (hazard ratio = 3.447, 95% CI: 1.931–6.154, *P* < 0.001) and TTR (hazard ratio = 3.010, 95% CI: 1.861–4.868, *P* < 0.001) (Additional file 1: Table S4).

These results established the clinical relevance of NOD2 in HCC and suggested that NOD2 may play a role in the development of HCC.

### Loss of NOD2 decreases hepatocarcinogenesis in mice

**Fig. 2:**
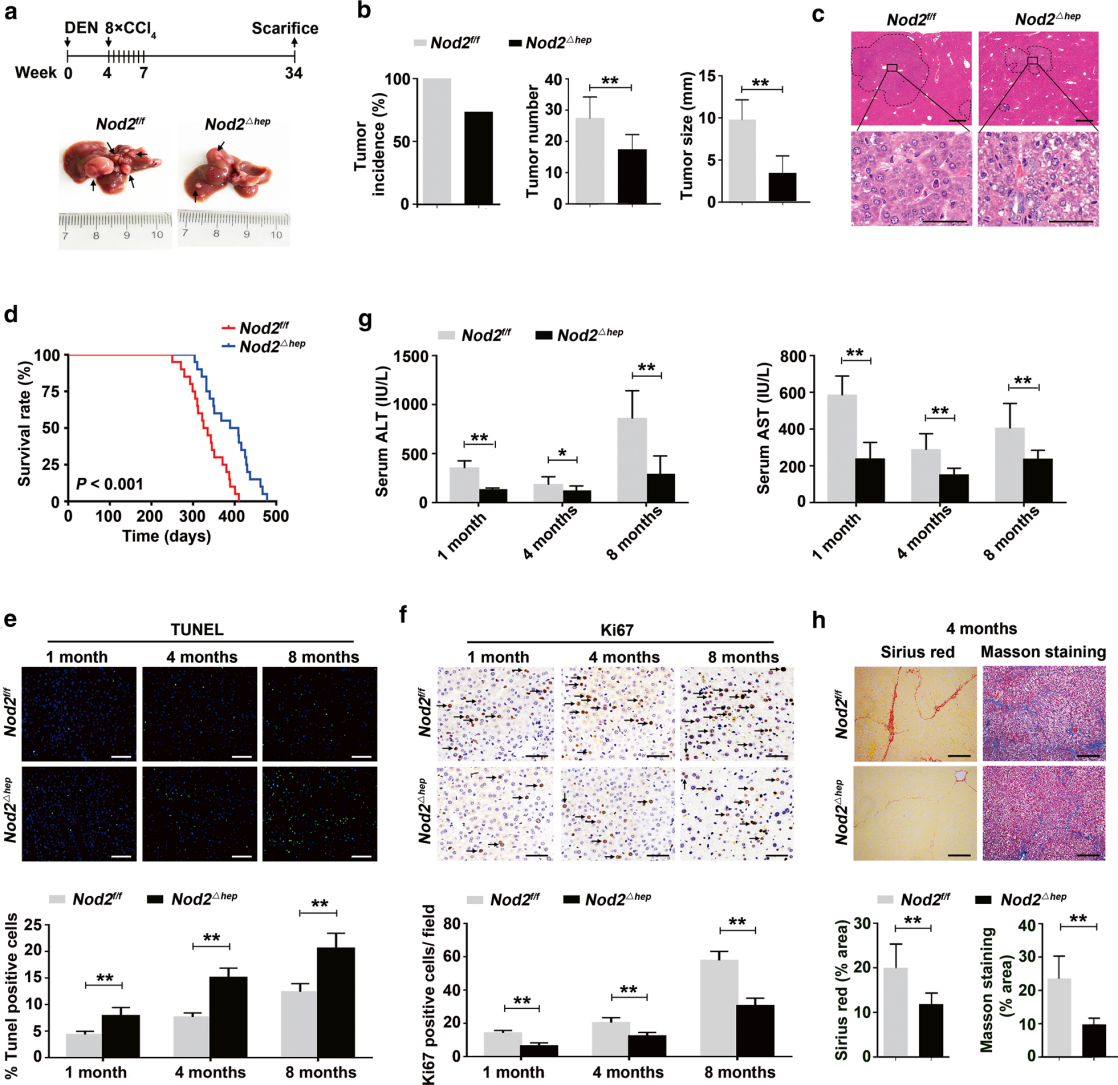
Hepatic NOD2 deficiency suppresses DEN/CCl_4_-induced hepatocarcinogenesis. **a** Effect of hepatic NOD2 deficiency on tumor growth in DEN/CCl_4_-induced HCC model. Male mice were injected with DEN (25 mg/kg, i.p.) at the age of 14–16 days followed by 8 injections of CCl_4_ (1.2 ml/kg, i.p., biweekly, starting 4 weeks after DEN injection) and killed 8 months after DEN injection. Representative gross appearance of the livers. Arrowheads indicate tumors in liver. **b** Quantification of tumor incidence in *Nod2*^*f/f*^ (*n* = 15) and *Nod2*^*△hep*^ (*n* = 15) mice (left panel). Quantification of tumor number (middle panel) and tumor size (right panel) in *Nod2*^*f/f*^ (*n* = 15) and *Nod2*^*△hep*^ (*n* = 11) mice. **c** Representative H&E staining of liver sections from *Nod2*^*f/f*^ and *Nod2*^*△hep*^. Scale bar, 200 μm (top panel) or 25 μm (bottom panel). **d** Survival rate of mice treated with DEN/CCl_4_. Median survival time for *Nod2*^*f/f*^ (*n* = 20) and *Nod2*^*△hep*^ (*n* = 20) mice was 330 and 399 days, respectively. **e–f** TUNEL (**e**) and Ki67 staining (**f**) in *Nod2*^*f/f*^ and *Nod2*^*△hep*^ mice livers. Arrowheads indicate Ki67-positive cells, *n* = 5–6. Scale bar, 100 μm (top panel) or 50 μm (bottom panel). **g** Serum ALT and AST levels in *Nod2*^*f/f*^ and *Nod2*^*△hep*^ mice. *n* = 6. **h** Sirius red and Masson staining in *Nod2*^*f/f*^ and *Nod2*^*△hep*^ mice livers 4 months after DEN injection. *n* = 6. Scale bar, 100 μm. Data were shown as mean ± SD, and significance was determined using ordinary one-way ANOVA with Sidak test (**e**,** f**), and unpaired Student’s t test (**b**,** g**,** h**). *P < 0.05, **P < 0.01

Next, we constructed hepatocyte-specific *Nod2*-knockout (*Nod2*^*△hep*^) mice (Additional file 3: Fig. S3) and studied the role of hepatic NOD2 in a DEN/CCl_4_ HCC model of *Nod2*^*f/f*^* and Nod2*^*△hep*^ mice. Eight months after DEN injection, all *Nod2*^*f/f*^ mice (15/15) developed liver tumors, whereas only 73.3% (11/15) of *Nod2*^*△hep*^ mice did. The number of tumor nodules was nearly 1.5-fold lower in *Nod2*^*△hep*^ mice than that in *Nod2*^*f/f*^ mice (17.36 ± 4.93 vs. 27.40 ± 6.98, *P* < 0.01). The maximal tumor diameters in *Nod2*^*△hep*^ mice were almost threefold lower than those in *Nod2*^*f/f*^ mice (3.41 ± 2.10 vs. 9.77 ± 2.42, *P* < 0.01) (Fig. 2a, b). Histopathological analysis revealed that liver tumors in *Nod2*^*△hep*^ and *Nod2*^*f/f*^ mice were basophilic and showed similar pathological characteristics (Fig. 2c). DEN/CCl_4_-treated *Nod2*^*△hep*^ mice had a higher survival rate than DEN/CCl_4_-treated *Nod2*^*f/f*^ mice (Fig. 2d). These results showed that loss of hepatic NOD2 attenuates the tumorigenesis of DEN/CCl_4_-induced HCC.

To determine whether NOD2 deletion affects HCC development at the cellular level, we measured cell death and proliferation in DEN/CCl_4_-treated livers. Compared to *Nod2*^*f/f*^ mice, *Nod2*^*△hep*^ mice displayed more apoptotic hepatocytes (Fig. 2e) and attenuated Ki67^+^ cells (Fig. 2f). The hepatocyte injury indicator serum alanine aminotransferase (ALT) and aspartate aminotransferase (AST) were lower in the serum of *Nod2*^*△hep*^ mice (Fig. 2g). The degree of liver fibrosis was also decreased in *Nod2*^*△hep*^ mice, indicated by Sirius Red and Masson staining 4 months after DEN/CCl_4_ treatment (Fig. 2h).

### Loss of hepatic NOD2 attenuates the inflammatory response, DNA damage and subsequent genomic instability during hepatocarcinogenesis

**Fig. 3:**
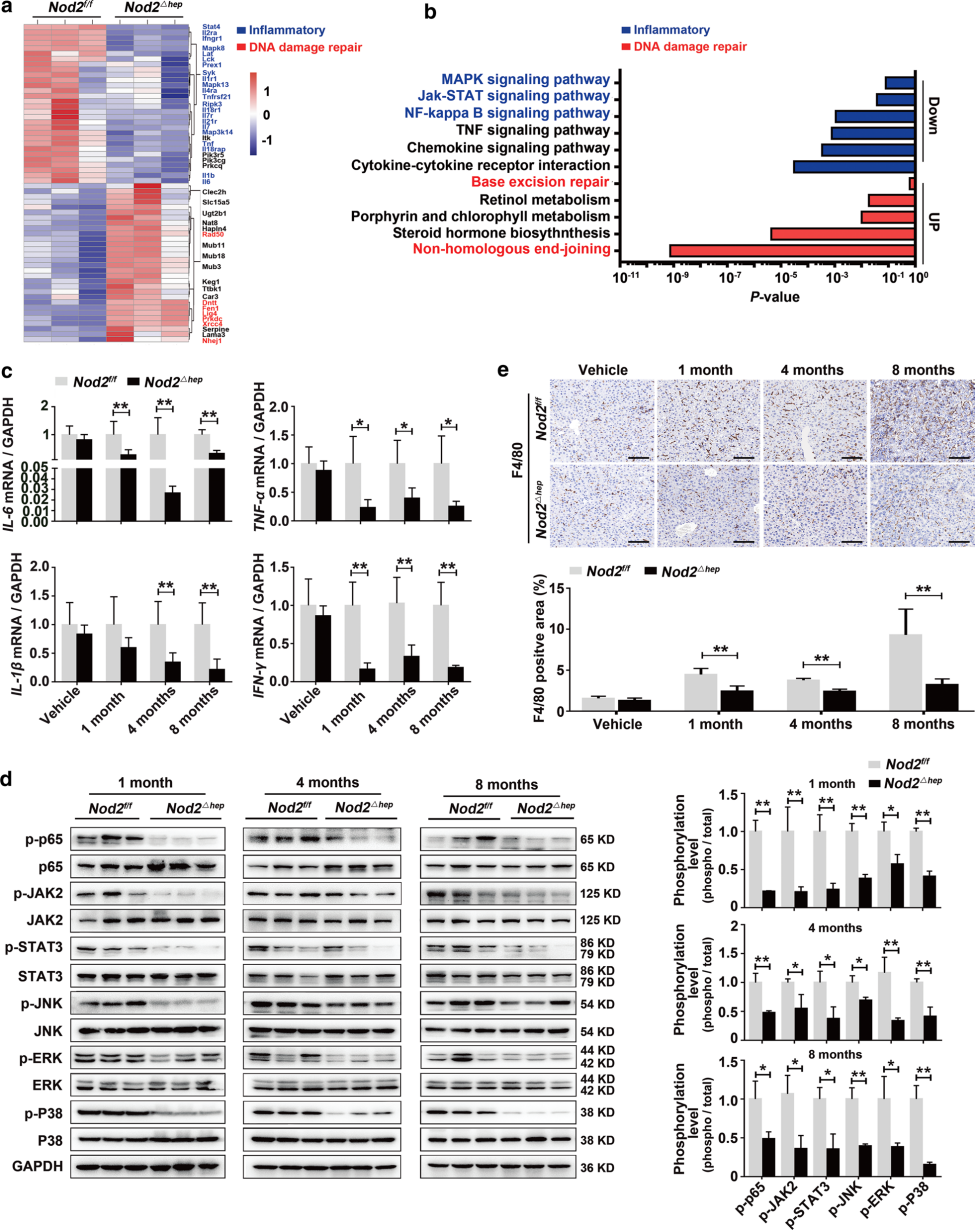
Hepatic NOD2 deficiency attenuates DEN/CCl_4_-induced liver inflammatory responses. **a**,** b** Hierarchical clustering (**a**) and KEGG pathway analysis (**b**) of up- and downregulated genes in livers from *Nod2*^*f/f*^ and *Nod2*^*△hep*^ mice treated with DEN/CCl_4_ for 4 months. **c** Relative mRNA expression of *IL-6, TNF-α, IL-1β* and *IFN-γ* in livers of *Nod2*^*f/f*^ and *Nod2*^*△hep*^ mice treated with DEN/CCl_4_ for the indicated time points, *n* = 6. **d** Western blots analysis of NF-κB (p65), JAK2/STAT3 and MAPK (ERK, JNK, P38) pathways in livers of *Nod2*^*f/f*^ and *Nod2*^*△hep*^ mice treated with DEN/CCl_4_ for the indicated time points. **e** Representative immunohistochemistry images and quantification of F4/80 in livers of *Nod2*^*f/f*^ and *Nod2*^*△hep*^ mice treated with DEN/CCl_4_ for the indicated time points, *n* = 5. Scale bar, 50 μm. Data were shown as mean ± SD, and significance was determined using unpaired Student’s t test (**c–e**). *P < 0.05, **P < 0.01

Chronic inflammatory response, DNA damage and subsequent genomic instability are hallmarks of carcinogens-induced HCC [18]. We performed RNA-seq analyses of livers from DEN/CCl_4_-treated *Nod2*^*f/f*^ and *Nod2*^*△hep*^ mice (*n* = 3, male) (SRA accession: PRJNA685328). Hierarchical clustering showed that multiple inflammatory cytokines (IL-6, TNF-α, IL-1, IFN-γ, etc.) were downregulated in *Nod2*^*△hep*^ livers, while genes involved in DNA damage repair upregulated, compared to *Nod2*^*f/f*^ livers (Fig. 3a and Additional file 1: Table S5). KEGG pathway enrichment analysis showed that genes downregulated in *Nod2*^*△hep*^ were enriched for the pro-inflammatory pathways (NF-κB, JAK-STAT and MAPK), while genes upregulated were enriched for DNA damage repair pathways (NHEJ and base excision repair), compared to *Nod2*^*f/f*^ livers (Fig. 3b).

Consistent with RNA-seq data, inflammatory cytokine genes (IL-6, TNF-α, IL-1β and IFN-γ), pro-inflammatory pathways (NF-κB (p65), JAK2/STAT3 and MAPK (ERK, JNK, P38), as well as ROS biosynthesis genes were positively co-enriched with NOD2 (Additional file 3: Fig. S4a, b) in GSEA analyses of the TCGA HCC dataset (*n* = 371) with the gene ontology (GO) and Hallmark gene sets. Reduced pro-inflammatory cytokine levels (IL-6, TNF-α, IL-1β and IFN-γ) and attenuated activation of pro-inflammatory pathways (NF-κB (p65), JAK2/STAT3 and MAPK (JNK, ERK, P38)) were further confirmed in DEN/CCl_4_-treated *Nod2*^*△hep*^ livers using qRT-PCR, ELISA and western blot analyses, respectively (Fig. 3c, d and Additional file 3: Fig. S5). Consistently, liver macrophages infiltration, as quantified with F4/80 staining, was diminished in *Nod2*^*△hep*^ compared to *Nod2*^*f/f*^ mice (Fig. 3e).

**Fig. 4:**
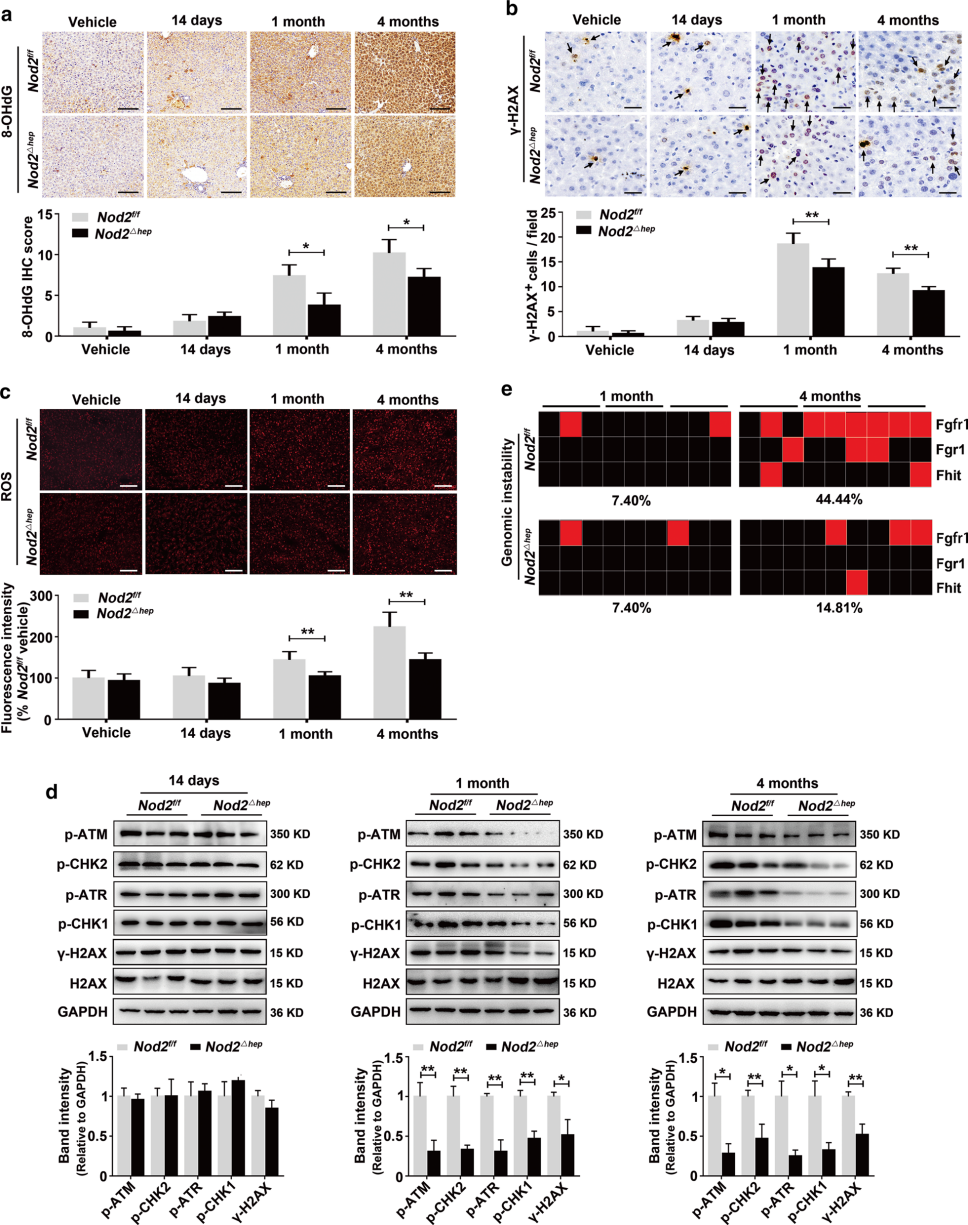
Hepatic NOD2 deficiency reduces DEN/CCl_4_-induced DNA damage. **a**,** b** Representative immunohistochemistry staining and semi-quantitative analysis of 8-OHdG (**a**) and γ-H2AX (**b**) in liver sections of *Nod2*^*f/f*^ and *Nod2*^*△hep*^ mice treated with DEN/CCl_4_ for the indicated time points. Arrowheads indicate γ-H2AX^+^ cells, *n* = 5. **c** Representative immunofluorescence images and quantification of ROS in liver of *Nod2*^*f/f*^ and *Nod2*^*△hep*^ mice treated with DEN/CCl_4_ for the indicated time points, *n* = 5. **d** Western blot analysis and quantification of ATM/CHK2, ATR/CHK1 pathways and γ-H2AX^+^ in livers of *Nod2*^*f/f*^ and *Nod2*^*△hep*^ mice treated with DEN/CCl_4_ for the indicated time points, *n* = 3. **e** Allelic imbalances (AI) were measured using TaqMan copy number assay in livers of *Nod2*^*f/f*^ and *Nod2*^*△hep*^ mice treated with DEN/CCl_4_ for the indicated time points. Each square represents one area of microdissected liver tissue, and lines indicate different areas of the same liver sample (red, AI; black, no AI). Data were shown as mean ± SD, and significance was determined using Student’s t test (**a–d**). *P < 0.05, **P < 0.01. Scale bar, 100 μm or 25 μm

In line with RNA-seq data, DNA damage recognition and repair pathways were negatively co-enriched with NOD2 in GSEA analyses of the TCGA HCC dataset (Additional file 3: Fig. S4c). Decreased 8-OHdG expression (a DNA damage marker) (Fig. 4a), γ-H2AX^+^ (a DNA double-strand break marker) cell numbers (Fig. 4b) and ROS levels (Fig. 4c) were observed in DEN/CCl_4_-treated *Nod2*^*△hep*^ livers, compared to those in DEN/CCl_4_-treated *Nod2*^*f/f*^ livers. Attenuated DDR pathways (ATM/CHK2, ATR/CHK1) activation and reduced γ-H2AX level were demonstrated with western blot analysis of DEN/CCl_4_-treated *Nod2*^*△hep*^ livers (Fig. 4d). Remarkably, DEN/CCl_4_-treated *Nod2*^*△hep*^ livers presented decreased genetic instability (14.81%) compared to DEN/CCl_4_-treated *Nod2*^*f/f*^ livers (44.44%) (Fig. 4e), as measured by allelic imbalances at chromosomal common fragile sites (CFS) using the TaqMan copy number assay [19].

These results collectively demonstrate that hepatic NOD2 knockout attenuates the inflammatory response and DNA damage in DEN/CCl_4_-induced HCC model.

### MDP, a NOD2 agonist, promotes hepatocarcinogenesis in mice

Increased intestinal permeability may allow the translocation of gut microbial metabolites such as MDP, into the liver via gut-liver axis in HCC patients. In line with this hypothesis, we observed an increased MDP level in HCC tissues, compared to that in normal liver tissue (Additional file 3: Fig. S6). To determine the role of MDP in hepatocarcinogenesis in vivo, we treated WT, *Nod2*^*△hep*^ and *Rip2*^*△hep*^ mice with MDP [20] in DEN/CCl_4_-induced HCC model (modeling procedures detailed in Additional file 3: Fig. S7a). Six months after DEN injection, compared with untreated group, MDP treatment significantly increased the tumor incidence (40.0% (6/15) vs. 80% (12/15)) and tumor burden (tumor number: 4.67 ± 0.82 vs. 10.83 ± 1.85, *P* < 0.01; tumor size: 7.12 ± 2.04 vs. 11.58 ± 2.13, *P* < 0.01), in the WT, but not in *Nod2*^*△hep*^ (tumor incidence: 27.8% (5/18) vs. 33.3% (5/15); tumor number: 2.2 ± 0.84 vs. 2.40 ± 1.14, *P* > 0.05; tumor size: 2.00 ± 0.71 vs. 3.60 ± 1.14, *P* > 0.05), indicating that MDP promotes tumorigenesis in an NOD2-dependent manner. Unexpectedly, MDP-induced increase of tumor incidence and burden was still observed in *Rip2*^*△hep*^ mice (tumor incidence: 46.7% (7/15) vs. 66.7% (10/15); tumor number: 4.00 ± 0.82 vs. 7.71 ± 1.25, *P* < 0.01; tumor size: 4.64 ± 1.11 vs. 8.00 ± 0.87, *P* < 0.01) (Additional file 3: Fig. S7b, c), although this increase was moderate, indicating that a RIP2-independent signal may be also involved in MDP-promoted hepatocarcinogenesis. Kaplan–Meier plot also showed that compared with untreated group, MDP treatment significantly decreased the survival rate in the WT (median survival time: 329 vs. 268 days) and *Rip*^*∆hep*^ (median survival time: 359 vs. 302 days), but only slightly decreased the survival rate in *Nod2*^*△hep*^ (median survival time: 398 vs. 378 days) in DEN/CCl_4_ HCC model.

### NOD2 activation enhances inflammatory response in a RIP2-dependent manner and exaggerates DNA damage in a RIP2-independent manner

It is well established that NOD2 activation drives inflammatory response via RIP2 binding and phosphorylation [9]. To determine whether the pro-inflammatory effect of NOD2 activation is mediated by RIP2 in the liver, we measured the expression of pro-inflammatory cytokines and macrophages infiltration in livers of mice from the MDP-treated HCC model. One month after DEN injection, MDP treatment significantly increased pro-inflammatory responses (expression of IL-6, TNF-α, IL-1β and IFN-γ, and the number of infiltrating macrophages) and DNA damage (8-OHdG and H2AX expression) in WT mice. NOD2 deficiency blocked MDP-exaggerated pro-inflammatory responses and DNA damage. In contrast, RIP2 deficiency only blocked MDP-potentiated inflammatory responses, but had no effect on MDP-increased DNA damage (Additional file 3: Fig. S7e–g), indicating a RIP2-dependent and a RIP2-independent mechanism in promoting pro-inflammatory responses and exaggerating DNA damage, respectively.

To determine the effect of NOD2 activation on DEN-induced inflammatory response in vitro*,* we treated primary hepatocytes with DEN alone or with DEN plus MDP (DEN/MDP). Consistent with our findings in vivo (Fig. 3a–d), MDP aggravated DEN-induced inflammatory response, evidenced by increased expression of IL-6, TNF-α, IL-1β and IFN-γ in hepatocytes (Additional file 3: Fig. S8a) and potentiated DEN-induced activation of NF-κB (p65), JAK2/STAT3 and MAPK (JNK, ERK,P38) (Additional file 3: Fig. S8b), which are well-known pro-inflammatory pathways downstream of RIP2 [9, 21]. In contrast, MDP did not enhance DEN-induced inflammatory response in hepatocytes from *Rip2*^*△hep*^ mice. Together, these findings indicated that, in a Rip2-dependent manner, NOD2 activation turns on the NF-κB, JAK2/STAT3 and MAPK signaling pathways to promote a pro-inflammatory response in the hepatocytes (Additional file 3: Fig. S8c).

We have found that *Rip2* knock-out had no effect on MDP-aggravated DNA damage in the DEN/CCl_4_ HCC model (Additional file 3: Fig. S7g). Similarly, when exposed to DEN in vitro, hepatocytes from *Rip2*^*△hep*^ mice still had aggravated DNA damage after MDP treatment, as determined by the activation of DDR pathways (ATM/CHK2, ATR/CHK1) and elevation of γ-H2AX level via western blots (Additional file 3: Fig. S9), further indicating that NOD2 activation in hepatocytes aggravates DEN-induced DNA damage via a RIP2-independent mechanism.

### NOD2 directly interacts with lamin A/C to promote its degradation through RIP2-independent nuclear autophagy

**Fig. 5:**
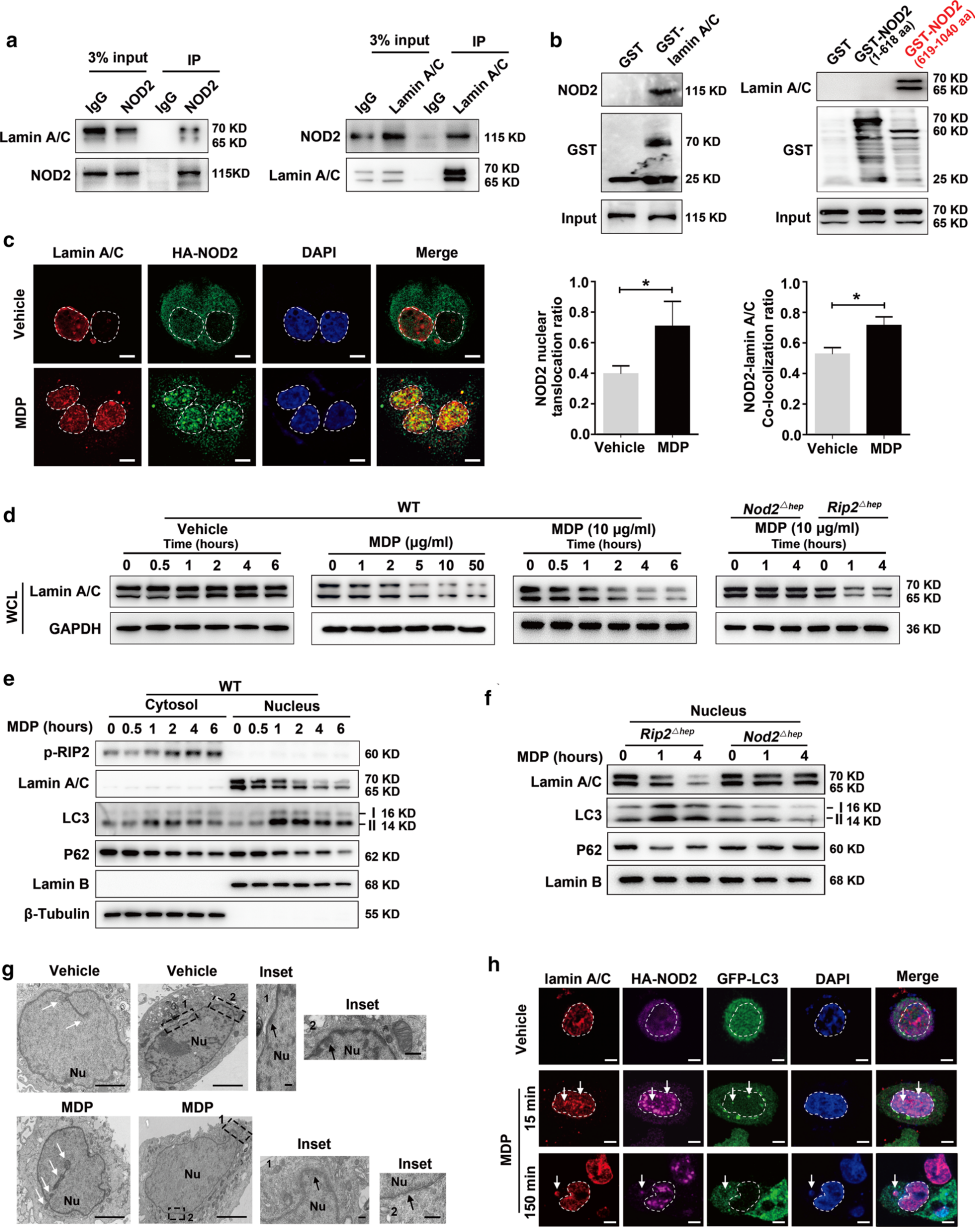
NOD2 interacts with lamin A/C and promotes its degradation through nuclear autophagy. **a** Co-immunoprecipitation of endogenous NOD2 with lamin A/C in primary hepatocytes. **b** GST pull-down assay showing that NOD2 directly interacts with lamin A/C through its LRR domain (amino acids 619–1040). **c** Immunofluorescence showed co-localization (yellow) of HA-NOD2 (green) and lamin A/C (red) in primary hepatocytes after MDP treatment. Bar graphs shown are HA-NOD2 nuclear translocation ratio and overlap co-efficient of HA-NOD2/lamin A/C co-localization, *n* = 3; *P < 0.05. Scale bar, 5 μm. **d** NOD2 activation decreases lamin A/C protein independently from RIP2. Primary hepatocytes from indicated mice were treated with different concentrations of MDP for 4 h or MDP (10 μg/ml) at the indicated time points. **e** Primary hepatocytes were treated with MDP (10 μg/ml), and then, p-RIP2, lamin A/C, LC3 and P62 protein levels were examined in the nucleus and cytoplasm. **f** Primary hepatocytes from indicated mice were treated with MDP (10 μg/ml). The protein levels of lamin A/C, LC3 and P62 were examined in the nucleus. **g** Transmission electron microscopy analysis of primary hepatocytes treated with MDP. White arrow: autophagosome; black arrow: perinuclear heterochromatin; representative nuclear membrane collapse and perinuclear heterochromatin decrease are labeled as event 1 and event 2, respectively. Nu, nucleus. Scale bar, 2.5 μm or 500 nm. **h** Primary hepatocytes expressing HA-NOD2 were infected with lentivirus carrying GFP-LC3 and treated with vehicle or MDP (10 μg/ml) for 2 h. Cells were then stained with lamin A/C and HA antibodies. co-localization (white) of lamin A/C (red), HA-NOD2 (purple), GFP-LC3 (green) and DAPI (blue) in primary hepatocytes was imaged by confocal microscopy after MDP treatment. Nuclear and cytoplasmic events are indicated by arrows. Scale bar, 5 μm. Data were shown as mean ± SD, and significance was determined using Student’s t test (**c**)

We have shown that NOD2 activation RIP2 independently aggravates DNA damage. To gain further insights into the mechanistic interplay between NOD2 and DNA damage, we used co-immunoprecipitation and liquid chromatography-tandem mass spectrometry (LC–MS/MS) to search for NOD2-interacting proteins and identified 28 potential candidates (Additional file 1: Table S6). Among them, nuclear matrix protein lamin A/C (*Lmna*) was identified by five unique peptides (Additional file 3: Fig. S10a, b). Interaction network analysis using RNA-seq data (*Nod2*^*△hep*^ and *Nod2*^*f/f*^ transcriptomes), String database (https://string-db.org/) and Cytoscape software suggested that *Lmna* might play a central role in the NOD2-aggravated DNA damage (Additional file 3: Fig. S10c). Bidirectional co-IP assays verified the physical interaction between endogenous NOD2 and lamin A/C in primary hepatocytes (Fig. 5a). GST pull-down assays showed that purified GST-NOD2 could directly interact with lamin A/C via NOD2 C‐terminal LRR domain (amino acids 619–1040) (Fig. 5b). Confocal immunofluorescence showed that, upon MDP treatment, cytoplasmic NOD2 underwent nuclear translocation and co-localized with lamin A/C (Fig. 5c).

Using the cNLS Mapper program (http://nls-mapper.iab.keio.ac.jp/cgi-bin/NLS_Mapper_form.cgi) [22], a bipartite nuclear localization sequences (NLS), _407_LRKFVRTECQLKGFSEEGIQLYLRKHHREP_436_, were identified on NOD2 (Additional file 3: Fig. S11a). Immunofluorescence and western blot studies showed that deletion of this NLS completely abolished the ability of NOD2 to translocate into the nucleus (Additional file 3: Fig. S11b, c).

To determine how NOD2 regulates lamin A/C, we measured the effect of NOD2 activation on lamin A/C protein level and found that MDP treatment concentration- and time-dependently decreased lamin A/C protein in primary hepatocytes, and that this effect is NOD2-dependent, but RIP2-independent, because NOD2 deficiency, but not RIP2 deficiency, could abolish this effect (Fig. 5d and Additional file 3: Fig. S12a). MDP treatment did not affect the mRNA level (Additional file 3: Fig. S12b), but decreased the protein stability of lamin A/C (Additional file 3: Fig. S12c). MDP-induced lamin A/C degradation was blocked by 3-MA (an autophagy inhibitor) (Additional file 3: Fig. S12d), but not by MG-132 (a proteasome inhibitor) (Additional file 3: Fig. S12e), indicating an autophagy-dependent pathway. In support of these results, we found that MDP treatment induced nuclear autophagy, evidenced by increased LC3-II and decreased p62 in the nucleus (Fig. 5e and Additional file 3: Fig. S12f). MDP-induced nuclear autophagy could be abolished by NOD2 deficiency, but not RIP2 deficiency (Fig. 5f and Additional file 3: Fig. S12g), indicating a NOD2-dependent, but RIP2-independent manner.

TEM demonstrated more nuclear autophagosomes (Fig. 5g and Additional file 3: Fig. S12h), increased nuclear membrane collapse and less perinuclear heterochromatin in MDP-treated primary hepatocytes, compared to vehicle (Fig. 5g), indicating that MDP triggers nuclear autophagy events. Consistently, we found that NOD2 interacted with ATG16L1, a protein governing autophagosome formation, in hepatocytes following MDP treatment (Additional file 3: Fig. S13). Confocal immunofluorescence analysis revealed that lamin A/C co-localized with GFP-LC3 in nucleus after 15 min of MDP stimulation (Fig. 5h, middle panel), while nuclear lamin A/C, HA-NOD2 and DAPI bodies appeared in cytoplasm and co-localized with GFP-LC3 after 150 min of MDP stimulation (Fig. 5h, bottom panel), indicating that lamin A/C is an autophagy substrate upon MDP stimulation.

Live-cell imaging on mCherry-GFP-lamin A/C-expressing hepatocytes showed that upon MDP stimulation, lamin A/C underwent a nucleus-to-cytoplasm transport process at 0–120 min, accompanied by nuclear membrane blebbing at 45 min. Lamin A/C gradually entered the lysosome at 135–165 min, as lamin A/C displayed cytoplasmic red-only bodies at 165 min in cells, and GFP signal was quenched by the acidic pH environment inside the lysosome [23] (Additional file 3: Fig. S14a). Super-resolution microscopy analysis further confirmed that lamin A/C was degraded by lysosome pathway, evidenced by co-localization of lamin A/C and Lyso-Tracker Red in cytoplasm of MDP-stimulated hepatocytes (Additional file 3: Fig. S14b).

Together, these findings show that NOD2 activation triggers a novel nuclear autophagy pathway to degrade lamin A/C protein (Additional file 3: Fig. S14c).

### NOD2 activation impairs DNA repair through lamin A/C degradation

**Fig. 6:**
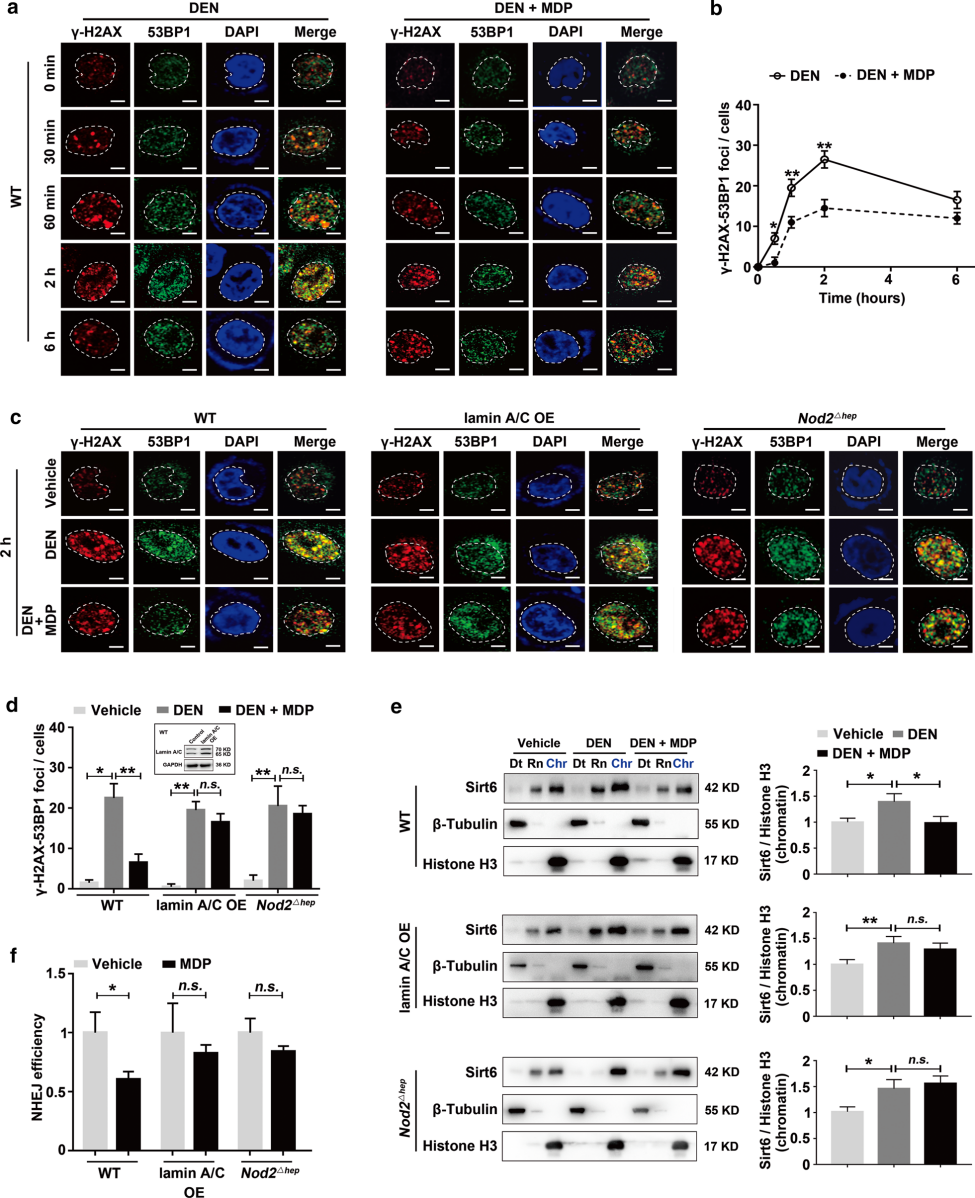
NOD2 activation inhibits DNA damage repair via lamin A/C degradation. **a**,** b** MDP (10 μg/ml) treatment inhibited 53BP1 recruitment to the DNA lesion sites in DEN-treated (100 μg/ml) hepatocytes. Representative images (**a**) and quantification (**b**) of γ-H2AX/53BP1 overlapping foci, *n* = 3. Immunofluorescence showed co-localization (yellow) of 53BP1 (green) and γ-H2AX (red). **c**,** d** The inhibitory effect of MDP (10 μg/ml) on 53BP1 recruitment is NOD2-dependent and can be reversed by lamin A/C overexpression (OE) in DEN-treated (100 μg/ml) hepatocytes. Representative images (**c**) and quantification (**d**) of γ-H2AX/53BP1 overlapping foci, *n* = 3. **e** MDP (10 μg/ml) treatment NOD2-dependently decreased chromatin-bound SITR6 in DEN-treated (100 μg/ml) hepatocytes, which was restored by lamin A/C overexpression. Cell lysates were fractionated to determine chromatin-bound Sirt6 levels. Bar graph shows the densitometric analyses of chromatin Sirt6 bands relative to Histone 3, *n* = 3. Dt, detergent-extractable fraction; Rn, RNase-extractable fraction; Chr, purified chromatin fraction, indicated in blue. **f** MDP (10 μg/ml) treatment NOD2-dependently decreased NHEJ efficiency, which was reversed by lamin A/C overexpression, *n* = 3. Data were shown as mean ± SD, and significance was determined using ordinary two-way (**b**) or one-way ANOVA with a Sidak test (**d**,** e**) and Student’s t test (**f**). *P < 0.05, **P < 0.01, *n.s.*, not significant. Scale bar, 5 μm

Lamin A/C plays important roles in DNA repair and genomic stability [24, 25], by promoting recruitment of 53BP1 to the damage foci and enrichment of SIRT6 to the chromatin upon DNA damage, thus facilitating DNA damage repair through NHEJ pathways [26–28]. Consistently, our Comet assay and double-labeled immunofluorescence of γ-H2AX/53BP1 showed that MDP treatment aggravated DEN-induced DNA damage (Additional file 3: Fig. S15) and reduced the number of γ-H2AX/53BP1-colocalized foci (Fig. 6a, b), respectively, in hepatocytes. Western blots of chromatin-bound fractionation and NHEJ assay showed that MDP treatment decreased the amount of chromatin-bound SITR6 (Fig. 6e) and diminished the activity of NHEJ pathway (Fig. 6f), respectively, in DEN-treated hepatocytes. All these effects were NOD2-dependent and could be restored by lamin A/C overexpression (Additional file 3: Fig. S15, Fig. 6c–f). Together, our results suggest that bacterial MDP reduces DNA repair and increases DNA damage through NOD2-lamin A/C axis.

To investigate whether lamin A/C is decreased in HCC, we next measured the lamin A/C protein level in the livers of DEN/CCl_4_-treated mice. Lamin A/C protein gradually decreased in WT mice, but not *Nod2*^*△hep*^ mice, during the progression of DEN/CCl_4_-induced HCC (Additional file 3: Fig. S16a). This decrease appeared to be NOD-dependent, because *Nod2*^*△hep*^ mice had higher lamin A/C level than *Nod2*^*f/f*^ mice (Additional file 3: Fig. S16b).

### NOD2 activation promotes hepatocarcinogenesis through RIP2 and lamin A/C

**Fig. 7:**
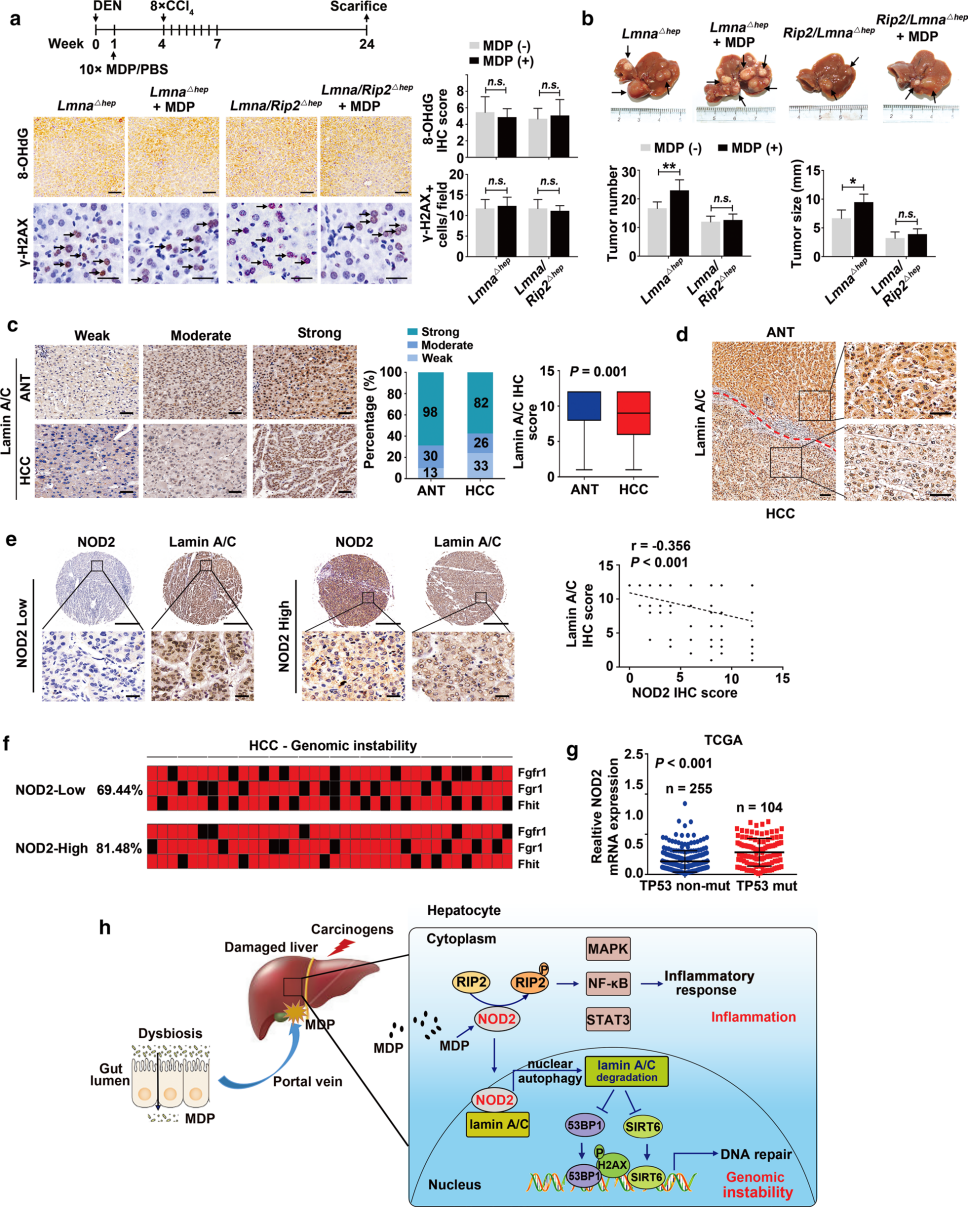
Both RIP2 and lamin A/C are required for NOD2 activation-promoted hepatocarcinogenesis. **a**,** b** Male *Lamn*^*△hep*^ and *Rip2/Lamn*^*△hep*^ mice were injected with DEN (25 mg/kg, i.p.) at the age of 14–16 days followed by 8 injections of CCl_4_ (1.2 ml/kg, i.p., biweekly, starting 4 weeks after DEN injection) and either MDP (100 μg/mice, every other day) or PBS via intraperitoneal injection for 10 times starting three week before the first injection of CCl_4_. Mice were killed 1 and 6 months after DEN injection. **a** Representative immunohistochemistry images and quantification of 8-OHdG and γ-H2AX in livers of *Lamn*^*△hep*^ and *Rip2/Lamn*^*△hep*^ mice 1 month after DEN treatment. Arrowheads indicate γ-H2AX^+^ cells, *n* = 5. Scale bar, 50 μm or 25 μm. **b** Representative gross appearance, quantification of tumor number and size 6 months after DEN treatment are shown, *n* = 5–8. Arrowheads indicate tumors in liver. **c** Immunohistochemistry staining and semi-quantification of lamin A/C in HCC samples from ZHH1. *n* = 141. Scale bar, 50 μm. **d** Representative immunohistochemistry images of lamin A/C in HCC and ANT tissues. Scale bar, 50 μm. **e** Negative correlation between NOD2 and lamin A/C protein in HCC samples from ZHH1, *n* = 141; Pearson correlation analysis. Scale bar, 400 μm or 50 μm. **f** Allelic imbalances were measured in HCC clinical samples (red, AI; black, no AI). **g** Increased NOD2 expression in HCC patients from TCGA database with TP53 mutations. **h** Proposed schematic of hepatic NOD2 regulatory mechanisms in HCC carcinogenesis. Data were shown as mean ± SD, and significance was determined using ordinary two-way ANOVA with Sidak test (**a**,** b**), paired (**c**) and unpaired Student’s t test (**g**). *P < 0.05, **P < 0.01, *n.s.*, not significant

To determine the role of lamin A/C in NOD2-promoted hepatocarcinogenesis in vivo, we treated *Lamn*^*△hep*^ and *Rip2/Lamn*^*△hep*^ mice with MDP in DEN/CCl_4_-induced HCC model. Compared with untreated group, MDP treatment cannot affect DNA damage (8-OHdG and H2AX expression) (Fig. 7a), but increased pro-inflammatory responses (expression of IL-6, TNF-α, IL-1β and IFN-γ, and the number of infiltrating macrophages) (Additional file 3: Fig. S17a, b), in lamin A/C^*△hep*^ mice. In addition, MDP treatment still moderately increased the tumor burden in lamin A/C^*△hep*^ mice (tumor number: 16.50 ± 2.43 vs. 22.75 ± 3.92, *P* < 0.01; tumor size: 6.58 ± 1.53 vs. 9.38 ± 1.51, *P* < 0.01) (Fig. 7b). Notably, hepatic double-knockout of *Lamin A/C* and *Rip2* abolished MDP-promoted hepatocarcinogenesis, evidenced by unchanged tumor burden (tumor number: 11.80 ± 2.17 vs. 12.40 ± 2.30, *P* > 0.05; tumor size: 3.10 ± 1.19 vs. 3.80 ± 1.04, *P* > 0.05), DNA damage and pro-inflammatory responses in *Rip2/Lamn*^*△hep*^ mice (Fig. 7a, b, Additional file 3: Fig. S17a, b).

Finally, to validate our findings in human samples, we analyzed the association between NOD2 and lamin A/C in the ZHH1 cohort (*n* = 141) with IHC. We found that, in contrast to the increased NOD2 level (Fig. 1c, 1), lamin A/C level was decreased in HCCs, compared to their matched ANTs (average score 8.84 ± 3.582 vs. 9.76 ± 2.957, *P* = 0.001; Fig. 7c, d). Notably, NOD2-high tumors showed low lamin A/C expression and vice versa. Pearson correlation analysis demonstrated a negative correlation between NOD2 and lamin A/C protein level in HCC (Fig. 7e).

In line with our findings that NOD2 deficiency decreases genetic instability in DEN/CCl_4_-treated HCC mice model (Fig. 4e), we found that NOD2-high HCCs had higher genetic instability than NOD2-low HCCs, as evidenced by a higher level of allelic imbalances (AI, 81.48% vs. 69.44%) at chromosomal common fragile sites (CFS) (Fig. 7f). Results were further confirmed in TCGA HCC cohort, in which we found a higher NOD2 expression in HCCs with mutated TP53 (a biomarker of genomic instability [29]) (Fig. 7g). Moreover, we also examined the mutation status of LMNA and TP53 in ZHH1 and TCGA HCC cohort. We found that LMNA presents a low mutation status in HCC [(2/141, 1.42%) in ZHH1; (5/359, 1.39%) in TCGA cohort]. LMNA-Mut groups showed increased TP53 mutation (1/2, 50% in ZHH1; 3/5, 60% in TCGA), compared with LMNA-Wt groups (24/139, 17.3%; 101/354, 28.53%), indicated that LMNA mutation could be associated with increased genomic instability. (Additional file 3: Fig. S18).

## Discussion

Genomic instability induced by chronic inflammation can be detected in 90% of early HCCs and dysplastic nodules in cirrhotic liver. For instance, telomerase reverse transcriptase (TERT) promoter mutations are presented in 25% of dysplastic nodules, increased in early HCC (61%) and remained stable in progressed and advanced HCC [30], which suggests that genetic instability might be an early hallmark in hepatocarcinogenesis. However, the mediators responsible for genetic instability in early HCC remain poorly understood. Our study reveals NOD2 as a novel hepatic mediator, sensing gut dysbiosis and linking gut-derived bacterial PAMPs with chronic inflammation and genomic instability in liver (summarized in Fig. 7h). We show that, clinically, NOD2 is overexpressed in HCC samples and closely correlates with poor prognosis of HCC patients. In DEN/CCl_4_-treated HCC model, hepatic NOD2 is activated and NOD2 deletion attenuates the tumorigenesis of HCC. Mechanically, in a RIP2-dependent manner, NOD2 activation triggers a pro-inflammatory response through activation of the NF-κB, JAK2/STAT3 and MAPK pathways. Remarkably, in a RIP2-independent way, activated NOD2 undergoes nuclear translocation and increases lamin A/C protein degradation via the nuclear autophagy pathway, consequently increasing genomic instability.

Usually, bacteria and their metabolites can damage host cell DNA directly by their genotoxins or indirectly by triggering inflammatory response [31]. Here, we found another approach whereby microbiota potentiates DNA damage through the disruption of the damage repair pathways. When DNA damage occurs, cells respond by activating DDR signal to recognize damaged DNA and recruiting DNA repair proteins to DNA damage sites to facilitate damage repair [2]. The disruption of the damage repair pathways may cause mutations or inactivation of certain oncogenes or tumor-suppressor genes, which can lead to the death of normal cells or transformation into tumor cells [2]. Consistence with this, we showed that persistent activation of hepatocyte NOD2 signaling by bacteria impairs DNA repair and increases carcinogenesis during the early stage of HCC development.

Notably, we discovered a novel mechanism linking NOD2 signaling to DDR through lamin A/C degradation. Lamin A/C, encoded by *LMNA* gene, is a nuclear matrix protein key in the maintenance of genome stability [32]. Mutations in *LMNA* are associated with premature aging syndromes and muscular dystrophies, collectively termed as laminopathy [33]. Patients with severe laminopathy exhibit nuclear abnormalities including genome instability, epigenetic dysregulation and telomere shortening, and die in their teens. Consistently, we observed that NOD2 activation RIP2 independently promotes lamin A/C protein degradation and consequently inhibits the repair of DEN-induced DNA damage, by decreasing the recruitment of 53BP1 to DNA damage sites, reducing SIRT6 enrichment to chromatin and diminishing the activity of NHEJ pathway. Supporting our finding, previous studies have reported that loss of lamin A/C increases DNA damage by suppression of these pathways [25, 26, 34]. Recently, a study by Liu et al. [35] showed that LMNA may be an oncogene in HCC development. Indeed, previous studies have indicated that LMNA expression may be a double-edged sword in tumor initiation and progression: increased lamin A/C levels could facilitate tumor cell proliferation and migration in HCC progression, while decreased lamin A/C could induce genomic instability in tumor initiation [32]. Thus, it is worth noting that the role of LMNA in cancer is probably context dependent and vary with stage of disease.

We found that NOD2 promotes lamin A/C protein degradation through nuclear autophagy pathway and identified NOD2 as a previously unknown initiator of nuclear autophagy. Previous studies have reported that NOD2 recruits ATG16L1 to the cell membrane at the bacterial entry site to kill the invading bacteria in human colon epithelial cells, macrophages and dendritic cells [36–38]. This antibacterial xenophage occurs in the cytoplasm, as a host defense machinery against pathogenic infections. We expanded these studies to hepatocytes and, for the first time, found that NOD2 activation triggers nuclear autophagy. To date, little is known about the role of nuclear autophagy in degradation of nuclear materials. A recent study reported that oncogenic stress-initiated nuclear autophagy mediates nuclear protein lamin B1 degradation, causing cell proliferation impairment, chromatin alteration and nuclear membrane disruption, and, eventually, driving cells senescence [23]. Our study shows that Gut-derived NOD2 agonist MDP could initiate nuclear autophagy and mediate lamin A/C degradation, which leads to nuclear matrix disruption, chromosomal aberration and accumulation of genomic mutations and instability, thereby promoting tumor formation. Thus, NOD2 appears to be an important PRR whereby microbial metabolites may cause nuclear DNA damage through the degradation of nuclear components.

Recent gut microbiome analyses showed that both gram (+) and gram (−) bacteria are enriched in cirrhosis patients and early HCC [39, 40]. In addition, oral treatment with gram (+)-specific antibiotics (vancomycin or ampicillin) and gram (−)-specific antibiotics (metronidazole and neomycin) was shown to significantly reduce liver inflammation and hepatocarcinogenesis [5]. Collectively, these studies indicated that gut-derived PAMPs from gram (+) and gram (−) bacteria could contribute to hepatocarcinogenesis. In this context, NOD2 may serve as a unique bridge between gut-derived microbiota and hepatocarcinogenesis, because it can sense MDP from both gram (+) and gram (−) bacteria. Supporting this hypothesis, our findings in clinic samples, DEN/CCl_4_-induced HCC mice models and primary hepatocytes treated with DEN, demonstrate that hepatic NOD2 links gut-derived microbiota with liver inflammation and DNA damage.

Recent studies demonstrated that several NOD2 inhibitors including the initial hit compound (GSK669) and its related analogues, GSK400 and GSK717, possess sub-micromolar in vitro activity [41]. These inhibitors appear to exhibit significant selectivity for inhibition of NOD2-mediated responses since they do not block any of the other IL-8/NF-κB inducing pathways including NOD1, TNFR1 and TLR2 [41]. Although NOD2 inhibitors have not yet been tested in the clinical trials, previous studies have reported that NOD2 inhibitors exhibited favorable anticancer activity in vivo [42, 43]. Therefore, NOD2 inhibitors may be promising chemical tools to treat HCC patients, when in combination with other therapies such as chemotherapeutics or immunotherapy.

## Conclusion

Here we provide evidence showing that NOD2, a general sensor of gram (+) and gram (−) bacteria, acts as a hepatic mediator linking the gut-derived bacterial PAMPs with chronic inflammation and the genomic instability in hepatocarcinogenesis. Clinically, NOD2 was overexpressed in HCC samples and closely correlated with poor prognosis of HCC patients. In our animal model, hepatic NOD2 was activated in the process of DEN/CCl_4_-induced HCC, while NOD2 deletion attenuated the tumorigenesis of DEN/CCl_4_-induced HCC. Mechanically, in a RIP2-dependent manner, NOD2 activation triggered a pro-inflammatory response through the activation of NF-κB, JAK2/STAT3 and MAPK pathways. Notably, we show that activated NOD2 acts as a previously unknown initiator of the nuclear autophagy pathway in a novel RIP2-independent manner, promoting the degradation of the nuclear component lamin A/C. A decrease in lamin A/C impairs the ability of hepatocytes to repair damaged DNA and, as a result, leads to increased genomic instability (summarized in Fig. 7h). Targeting hepatic NOD2 might represent a promising therapeutic strategy for HCC.

### Limitations of study

Our study still has limitation that the mechanism for a higher expression of NOD2 in HCC is still unclear. Previous studies have demonstrated that NOD2 promotor contains two NF-κB binding sites that enable the transcriptional activation of NOD2, following stimulation with TNF-α, which resulted in increased NOD2 mRNA and protein level [44–46]. Moreover, this up-regulation is augmented by IFN-γ [44]. We estimated that up-regulation of NOD2 expression might be part of a positive regulatory loop induced by inflammatory cytokines or bacterial components, which needs the further investigation.

## Supplementary Information

**Additional file 1:** **Online Table 1**. The primary antibodies and dilutions used in the study. **Online Table 2**. Primer sequences for qPCR, genotyping and plasmid construction in the study. **Online Table 3**. Correlation between NOD2 and clinicopathologic characteristics. **Online Table 4**. Univariate and multivariate analyses of prognostic factors associated with survival and recurrence (n = 141). **Online Table 5**. Down-regulated and up-regulated genes in livers of Nod2△hep vs. Nod2f/f mice. **Online Table 6**. Summary of mass spectrometry (MS) analysis of NOD2 interactors.

**Additional file 2.** Supplementary Materials and Methods.

**Additional file 3:** **Online Fig. 1**. NOD2 expression increases during the progression of DEN/CCl4- induced HCC, accompanied by an increase of p-RIP2 expression. **Online Fig. 2**. Receiver operating characteristic (ROC) curve analysis of NOD2 immunohistochemistry staining. **Online Fig. 3**. Generation of hepatocyte-specific Nod2-knockout mice (Nod2△hep). **Online Fig. 4**. Gene set enrichment analysis (GSEA) of the correlation between NOD2 expression and inflammation, as well as DNA damage in patients with HCC. **Online Fig. 5**. Hepatic NOD2 deficiency reduces DEN/CCl4-induced liver inflammatory response. **Online Fig. 6**. Increased MDP concentration in HCC tissues. **Online Fig. 7**. NOD2 agonist MDP promotes hepatocarcinogenesis. **Online Fig. 8**. NOD2 activation increases DEN/CCl4-induced inflammatory response in primary mouse hepatocytes. **Online Fig. 9**. NOD2 activation increases the activation of DNA damage response (DDR) pathway, and the level of γ-H2AX, in DEN-treated mouse hepatocytes, via an RIP2-independent manner. **Online Fig. 10**. The role of NOD2/lamin A/C interaction network in DNA damage repair process. **Online Fig. 11**. NOD2 harbors a nuclear localization sequence (NLS). **Online Fig. 12**. NOD2 activation regulates the protein stability of lamin A/C, but not the transcription of lamin A/C, through nuclear autophagy. **Online. Fig. 13**. NOD2 interacts with ATG16L1, a component of the LC3-conjugation complex. **Online Fig. 14**. Live-cell imaging analysis of mCherry-GFP-lamin A/C in hepatocytes. **Online Fig. 15**. Lamin A/C overexpression rescues the aggravating effect of NOD2 activation on DEN-induced DNA damage in hepatocytes. **Online Fig. 16**. Lamin A/C expression decreases during the progression of DEN/CCl4-induced HCC. **Online Fig. 17**. MDP increases DEN/CCl4-induced inflammatory response in Lamn△hep, but not in Rip2/Lamn△hep mice. **Online Fig. 18**. LMNA mutation associated with increased genomic instability in HCC.

## Data Availability

All data generated or analyzed during this study are included in this published article (and its supplementary information files).

## Abbreviations

HCC: Hepatocellular carcinoma
DDR: DNA damage response
PAMP: Pathogen-associated molecular pattern
DEN: Diethylnitrosamine
NOD2: Nucleotide-binding oligomerization domain 2
PRR: Pattern recognition receptor
NOD2: NOD-like receptor
LRR: Leucine-rich repeat
NATCH: Central nucleotide-binding domain
CARD: Caspase activation and recruitment domain
TLR4: Toll-like receptor 4
MDP: Muramyl dipeptide
ZHH: Zhongshan Hospital HCC
OS: Overall survival
TTR: Time to recurrence
3-MA: 3-Methyladenine
ATCC: American Type Culture Collection
TMA: Tissue microarray
IHC: Immunohistochemistry
ROC: Receiver operating characteristic
AUC: Area under the curve
TCGA: The Cancer Genome Atlas
GSEA: Gene Set Enrichment Analysis
SgRNA: Single-guide RNA
WT: Wild-type
ANOVA: Analysis of variance
SD: Standard deviation
Co-IP: Co-immunoprecipitation
MS: Mass spectrometry
TEM: Transmission electron microscope
NHEJ: Non-homologous end joining
ANT: Adjacent non-tumor
AFP: Alpha fetoprotein
ALT: Alanine aminotransferase
AST: Aspartate aminotransferase
GO: Gene ontology
CFS: Common fragile sites
LS-MS/MS: Liquid chromatography-tandem mass spectrometry
NLS: Nuclear localization sequences
TERT: Telomerase reverse transcriptase
