# Supplementary material for: Hepatic NOD2 promotes hepatocarcinogenesis via a RIP2-mediated proinflammatory response and a novel nuclear autophagy-mediated DNA damage mechanism
Source: J Hematol Oncol. 2021 Jan 7;14:9. doi: 10.1186/s13045-020-01028-4 (PMC7791875; doi:10.1186/s13045-020-01028-4)
Supplement: Supplementary file 1 — Additional file 1: Online Table 1. The primary antibodies and dilutions used in the study. Online Table 2. Primer sequences for qPCR, genotyping and plasmid construction in the study. Online Table 3. Correlation between NOD2 and clinicopathologic characteristics. Online Table 4. Univariate and multivariate analyses of prognostic factors associated with survival and recurrence (n = 141). Online Table 5. Down-regulated and up-regulated genes in livers of Nod2△hep vs. Nod2f/f mice. Online Table 6. Summary of mass spectrometry (MS) analysis of NOD2 interactors. [file 13045_2020_1028_MOESM1_ESM.docx]

**Online Table 1. The primary antibodies and dilutions used in the study.**

| **Antibodies** | **Dilution for WB** | **Dilution for IHC** | **Dilution for IF** | **Manufacturer** |
| --- | --- | --- | --- | --- |
| NOD2 | 1:500 | 1:50 | / | Atlas |
| NOD2 | 1:1000 | 1:100 | / | Abcam |
| RIP2 | 1:1000 | / | / | CST |
| p-RIP2 (ser176) | 1:1000 | 1:200 | / | Biorbyt |
| Ki67 | / | 1:200 | / | Abcam |
| F4/80 | / | 1:100 | / | CST |
| P38 | 1:1000 | / | / | CST |
| p-P38 (Thr180/Tyr182) | 1:1000 | / | / | CST |
| ERK | 1:1000 | / | / | CST |
| p-ERK (Thr202/Tyr204) | 1:1000 | / | / | CST |
| JNK | 1:1000 | / | / | CST |
| p-JNK (Thr183/Tyr185) | 1:1000 | / | / | CST |
| JAK2 | 1:1000 | / | / | CST |
| p-JAK2 (Tyr1008) | 1:1000 | / | / | CST |
| STAT3 | 1:1000 | / | / | CST |
| p-STAT3 (Tyr705) | 1:1000 | / | / | CST |
| P65 | 1:1000 | / | / | CST |
| p-P65 (ser536) | 1:1000 | / | / | CST |
| 8-OHdG | / | 1:50 | / | Santa Cruz |
| γ-H2AX (phospho s139) | 1:1000 | 1:200 | 1:200 | Abcam |
| H2AX | 1:1000 | / | / | CST |
| ATM (phosphor s1981) | 1:10000 | / | / | Abcam |
| p-ATR (ser428) | 1:1000 | / | / | CST |
| p-CHK2 (Thr68) | 1:1000 | / | / | CST |
| p-CHK1 (ser317) | 1:1000 | / | / | CST |
| Lamin A/C | 1:2000 | 1:200 | 1:200 | Proteintech |
| Lamin A/C | / | 1:350 | / | Abcam |
| HA | 1:1000 | / | 1:100 | CST |
| GST | 1:1000 | / | / | CST |
| LC3B | 1:1000 | / | / | Abcam |
| P62 | 1:1000 | / | / | Abcam |
| 53BP1 | / | / | 1:100 | Santa Cruz |
| Sirt6 | 1:1000 | / | / | CST |
| ATG16L1 | 1:1000 | / | / | CST |
| Histone3 | 1:1000 | / | / | CST |
| Lamin B | 1:1000 | / | / | ABclonal |
| β-Tubulin | 1:1000 | / | / | CST |
| GAPDH | 1:1000 | / | / | Beyotime |

**Online Table 2. Primer sequences for qPCR, genotyping and plasmid construction in the study.**

| **Gene name** | **Forward 5’-3’** | **Reverse 5’-3’** |
| --- | --- | --- |
| **qPCR** |  |  |
| Human GAPDH | AACAGCCTCAAGATCATCAG | AGTCCTTCCACGATACCAA |
| Human NOD2 | TGGTTCAGCCTCTCACGATGA | AGGACACTCTCGAAGCCTT |
| Mouse IL-6 | TCCATCCAGTTGCCTTCTTG | TTCCACGATTTCCCAGAGAAC |
| Mouse TNFα | TGCCTATGTCTCAGCCTCTTC | GGTCTGGGCCATAGAACTGA |
| Mouse IL-1β | TCGCTCAGGGTCACAAGAAA | CATCAGAGGCAAGGAGGAAAAC |
| Mouse IFN-γ | ATGAACGCTACACACTGCATC | CCATCCTTTTGCCAGTTCCTC |
| Mouse GAPDH | TGTGTCCGTCGTGGATCTGA | TTCGTGTTGAAGTCGCAGGAG |
| Mouse lmna | ACCCCGCTGAGTACAACCT | CTCTCGCTGCTTCCCGTTATC |
|  |  |  |
| **genotyping** |  |  |
| Nod2 | CAGGGCTCCATAGCCATCAGCT | CCACCTTCAAAGTACCAGAATCAAGC |
| Lmna | AGCTTTGTCTACTTTAAGGGTAGGGTG | TTGCCTATGTGGGACTTTGAGG |
| Alb-Cre | TGGCAAACATACGCAAGGG | CGGCAAACGGACAGAAGCA |
| Rip2 common | TTGGAGCTTCCTCTAGTGCTG |  |
| Rip2 wt |  | TGCAAAGTGATGTGACTGAATG |
| Rip2 mutant |  | CCTTCTATCGCCTTCTTGACG |
|  |  |  |
| **Plasmid construction** | | |
| HA-Nod2 | Fwd: CTACCGGACTCAGATCTCGAGGCCACCATGTACCCATACGACGTCCCAGACTACGCTTGCTCACAGGAAGAGTTCCAGG | |
|  | Rev: GTACCGTCGACTGCAGAATTCTCACAACAAGAGTCTGGCGTCC | |
| Lmna | Fwd: CTACCGGACTCAGATCTCGAGGCCACCATGGAGACCCCGTCACAGC | |
|  | Rev: GTACCGTCGACTGCAGAATTCTTACATGATGCTGCAGTTCTGG | |
| GFP-[Map1lc3a](https://www.ncbi.nlm.nih.gov/gene/66734) | Fwd: CTACCGGACTCAGATCTCGAGGCCACCATGCCCTCCGACCGGCCT | |
|  | Rev: CATCCTTCCCTCGATGGATCCGAAGCCGAAGGTTTCTTGGGA | |
| GFP-Nod2 | Fwd: CTACCGGACTCAGATCTCGAGGCCACCATGTGCTCACAGGAAGAGTTCCAGG | |
|  | Rev: CGCGTGTCGACTGCAGAATTCTCACAACAAGAGTCTGGCGTCC | |
| GFP-Nod2 (△NLS) | Primer1 Fwd: CTACCGGACTCAGATCTCGAGGCCACCATGTGCTCACACAGGAAGAGTTCCAGG | |
|  | Primer1 Rev: CGCGTGTCGACTGCAGAATTCTCACAACAAGAGTCTGGCGTCC | |
|  | Primer2 Fwd: CGGATGCTGTGTCAGCGCTCGGGGTGGCAGACCGCCTCAT | |
|  | Primer2 Rev: ATGAGGCGGTCTGCCACCCCGAGCGCTGACACAGCATCCG | |
|  | | |
| **Sanger sequencing** | | |
| LMNA-E04up | Fwd: GGACCTATTAGAGCCTTTGCC | |
|  | Rev: AGCCACCTGGGCGAACTCAC | |
| LMNA-E07 | Fwd: CCTGGCACTGTCTAGGCACA | |
|  | Rev: GGACAGGTGAATGGCTCTGAA | |
| LMNA-E08+9+10 | Fwd: CTCCTGGATTCAAGCGATTCTT | |
|  | Rev: ACTCCACATCCTGCGACCCT | |
| LMNA-E11+12 | Fwd: CCAGTTGCCAGCCAAGACTATGT | |
|  | Rev: CATACGCAGCACCAGGCACA | |
| LMNA-E13+14 | Fwd: GGCCTTTGAGCAAGATACACC | |
|  | Rev: CCTCACTGTCTAACTTCCACCC | |
| LMNA-E15 | Fwd: GAGGACAGAGTAAGCAGCAGG | |
|  | Rev: CCTGTTCAAGGTATAGGGAGGA | |
| LMNA-E16 | Fwd: CTGGCTCCTTGGGCACAGAA | |
|  | Rev: AGGATGCAGAGGTGGGCTGT | |
| LMNA-E17 | Fwd: CCCGAACCCAGGTGAGTTGT | |
|  | Rev: AGGTGAGGAGGACGCAGGAA | |
| LMNA-E08+9 | Fwd: TGACCCCTTTTCCTCATCT | |
|  | Rev: GTTGGGCATCACTGCTACA | |
| LMNA-E10 | Fwd: CGACTGGTGGAGATTGACA | |
|  | Rev: CCAAAGCCCTGAGAAGTGA | |
| LMNA-E11 | Rev: GGAGCCTACCTCATTGGACTT | |
| LMNA-E12 | Fwd: CCCCACTTGGTCTCCCTCT | |
|  | Rev: GGATAATAGCCATGATCTGTG | |
|  |  | |
| P53-E02-4 | Fwd: AAGTCTTGGGGATTTAGTGGT | |
|  | Rev: AGAGATGCTGAGGGTGTGAT | |
| P53-E05+6 | Fwd: AGACGCCAACTCTCTCTAGC | |
|  | Rev: CATTTACTTTGCACATCTCATG | |
| P53-E07 | Fwd: TTGCCACAGGTCTCCCCAAG | |
|  | Rev: GGACAGGCACAAACACGCAC | |
| P53-E08+9 | Fwd: ACAAGGGTGGTTGGGAGTAG | |
|  | Rev: CGGCATTTTGAGTGTTAGAC | |
| P53-E10 | Fwd: TGTTGCTTTTGTACCGTCAT | |
|  | Rev: GTATCCACACTCGTCCCTGG | |
| P53-E11 | Fwd: CATCTTGATTTGAATTCCCGT | |
|  | Rev: TGTCCTACTCCCCATCCTCC | |
| P53-E02-4S | Fwd: GGAAGTCCCTCTCTGATTGT | |
|  | Rev: AGGAAGCCAAAGGGTGAAGA | |
| P53-E05+6S | Rev: TGGGGTTATAGGGAGGTCAA | |
| P53-E07S | Rev: GGTGGATGGGTAGTAGTATG | |
| P53-E08+9S | Fwd: GGAGTAGATGGAGCCTGGT | |
| P53-E10S | Rev: TTCTGTGGATACACTGAGGC | |
| P53-E11S | Rev: GCTTCTGACGCACACCTATT | |

**Online Table 3. Correlation between NOD2 and clinicopathologic characteristics.**

|  | **NOD2 expression in tumor tissue** | | |
| --- | --- | --- | --- |
| **Clinical and pathological indexes** | **Low** | **High** | ***P* value** |
| Patients | 83 | 58 |  |
| Gender |  |  |  |
| Female | 15 | 7 | 0.334 |
| Male | 68 | 51 |  |
| Age (years) |  |  |  |
| < 50 | 29 | 20 | 0.955 |
| ≥ 50 | 54 | 38 |  |
| Hepatitis B surface antigen |  |  |  |
| Negative | 19 | 12 | 0.756 |
| Positive | 64 | 46 |  |
| Liver cirrhosis |  |  |  |
| No | 25 | 7 | **0.012** |
| Yes | 58 | 51 |  |
| Tumor encapsulation |  |  |  |
| No | 37 | 26 | 0.977 |
| Complete | 46 | 32 |  |
| Vascular invasion |  |  |  |
| No | 56 | 26 | **0.007** |
| Yes | 27 | 32 |  |
| Tumor number |  |  |  |
| Single | 71 | 37 | **0.003** |
| Multiple | 12 | 21 |  |
| Tumor size (cm) |  |  |  |
| < 5 | 51 | 25 | **0.032** |
| ≥ 5 | 32 | 33 |  |
| Edmondson grade |  |  |  |
| I-II | 43 | 45 | **0.002** |
| III-IV | 40 | 13 |  |
| BCLC stage |  |  |  |
| A | 47 | 22 | **0.029** |
| B/C | 36 | 36 |  |
| ALT (units/L) |  |  |  |
| < 75 | 73 | 47 | 0.256 |
| ≥ 75 | 10 | 11 |  |
| TB,μmol/L |  |  |  |
| < 20 | 78 | 56 | 0.765 |
| ≥ 20 | 5 | 2 |  |
| Albumin,g/l |  |  |  |
| < 35 | 5 | 4 | 1.000 |
| ≥ 35 | 78 | 54 |  |
| PT, s |  |  |  |
| < 13 | 76 | 53 | 1.000 |
| ≥ 13 | 7 | 5 |  |
| AFP, ng/mL |  |  |  |
| < 20 | 46 | 20 | **0.014** |
| ≥ 20 | 37 | 38 |  |

AFP, [alpha fetoprotein](http://www.iciba.com/alpha_fetoprotein); ALT, alanine aminotransferase; BCLC, Barcelona Clinic Liver Cancer; PT, prothrombin time; TB, total bilirubin. Bold values indicate *P* < 0.05, *P* values from χ^2^ test.

**Online Table 4. Univariate and multivariate analyses of prognostic factors associated with survival and recurrence (n = 141).**

| **Variables** | **OS** | | | |  | **TTR** | | | |
| --- | --- | --- | --- | --- | --- | --- | --- | --- | --- |
|  | Univariate | Multivariate | | |  | Univariate | Multivariate | | |
|  | *P* | HR | 95%CI | *P* |  | *P* | HR | 95%CI | *P* |
| NOD2 in tumor tissue (high vs. low) | **<0.001** | 3.447 | 1.931-6.154 | **<0.001** |  | **<0.001** | 3.010 | 1.861-4.868 | **< 0.001** |
| Gender (male vs. female) | 0.611 |  |  | NA |  | 0.948 |  |  | NA |
| Age, years (≥ 50 vs. < 50) | 0.907 |  |  | NA |  | 0.631 |  |  | NA |
| HBsAg(positive vs. negative) | 0.088 |  |  | NA |  | 0.934 |  |  | NA |
| Liver cirrhosis (yes vs. no) | **0.011** | 2.075 | 0.951-4.527 | 0.067 |  | 0.082 |  |  | NA |
| Tumor encapsulation (yes vs. none) | 0.521 |  |  | NA |  | 0.846 |  |  | NA |
| Vascular invasion (yes vs. no) | **< 0.001** | 2.023 | 1.150-3.559 | **0.014** |  | **< 0.001** | 2.698 | 1.658-4.391 | **<0.001** |
| Tumor number (multiple vs. single) | **<0.001** | 2.058 | 1.168-3.625 | **0.013** |  | **< 0.001** | 2.218 | 1.379-3.566 | **0.001** |
| Tumor size (cm) (≥5 vs. <5) | **<0.001** | 1.743 | 0.761-3.994 | 0.189 |  | **0.007** | 1.275 | 0.629-2.581 | 0.500 |
| Edmondson grade (III-IV vs. I-II) | **0.006** | 0.411 | 0.216-0.785 | **0.007** |  | **0.032** | 0.667 | 0.405-1.100 | 0.112 |
| BCLC stage (B/C vs.0-A) | **<0.001** | 1.595 | 0.647-3.930 | 0.310 |  | **0.001** | 1.203 | 0.567-2.553 | 0.630 |
| ALT,U/L (≥75 vs.< 75) | **0.023** | 1.665 | 0.841-3.295 | 0.143 |  | 0.143 |  |  | NA |
| TB,μmol/L (≥ 20 vs.< 20) | 0.647 |  |  | NA |  | 0.544 |  |  | NA |
| Albumin,g/L (≥35 vs.< 35) | 0.509 |  |  | NA |  | 0.799 |  |  | NA |
| PT, s (≥13 vs.<13) | 0.443 |  |  | NA |  | 0.669 |  |  | NA |
| AFP, ng/mL (≥ 20 vs.< 20) | **0.007** | 1.558 | 0.907-2.677 | 0.108 |  | **0.009** | 1.203 | 0.746-1.942 | 0.448 |

**Abbreviations:** HBsAg, hepatitis B surface antigen; AFP, α-fetoprotein; BCLC, Barcelona Clinic Liver Cancer; OS, overall survival; PT, prothrombin time; TTR, time to recurrence; TB, total bilirubin; HR, hazard ratio; 95%CI, 95% confidential interval; NA, not applicable. Bold values indicate *P* < 0.05. *P* value < 0.05 was considered statistically significant. Univariate analysis was calculated by the Kaplan–Meier method (log-rank test). Multivariate analysis was done using the Cox multivariate proportional hazard regression model with stepwise manner.

**Online Table 5.**Down-regulated and up-regulated genes in livers of *Nod2^△hep^* vs. *Nod2^f/f^* mice*.*

| **Gene symbols** | **Nod2^f/f^ 1** | **Nod2^f/f^ 2** | **Nod2^f/f^ 3** | *Nod2^△hep^* 1 | *Nod2^△hep^* 2 | *Nod2^△hep^* 3 | Upregulated (1)；Downregulated (-1) |
| --- | --- | --- | --- | --- | --- | --- | --- |
| Stat4 | 0.68972 | 0.80704 | 0.67615 | 0.08185 | 0.14532 | 0.1212 | -1 |
| Syk | 5.55975 | 1.6084 | 4.96338 | 0.77842 | 3.10372 | 1.90772 | -1 |
| Lck | 2.49673 | 2.97503 | 4.49541 | 0.28968 | 1.70034 | 0.81961 | -1 |
| Lat | 1.12448 | 2.03042 | 2.65739 | 0.11875 | 0.29648 | 0.6488 | -1 |
| Ifngr1 | 19.4458 | 16.1294 | 18.9982 | 2.35663 | 3.8815 | 1.9496 | -1 |
| Tnf | 4.32578 | 1.81202 | 2.99832 | 0.41045 | 0.633 | 0.16533 | -1 |
| 18r1 | 1.85727 | 0.29808 | 0.66701 | 0.11122 | 0.10154 | 0.14608 | -1 |
| IL18rap | 1.22783 | 0.49974 | 1.34629 | 0.11494 | 0.18249 | 0.06472 | -1 |
| IL1b | 9.6576 | 4.34626 | 6.07828 | 1.07066 | 0.70459 | 1.62426 | -1 |
| IL1r1 | 35.4228 | 5.68323 | 46.417 | 3.37268 | 15.8466 | 15.63 | -1 |
| IL21r | 3.64366 | 1.02308 | 2.10264 | 0.33339 | 0.40947 | 0.24143 | -1 |
| IL2ra | 1.10233 | 0.94999 | 1.07943 | 0.08916 | 0.1018 | 0.0375 | -1 |
| IL4ra | 17.4055 | 4.54673 | 11.6099 | 2.31576 | 8.32208 | 7.21355 | -1 |
| IL6 | 0.38387 | 0.22305 | 0.26552 | 0.04136 | 0.12109 | 0.09881 | -1 |
| IL7 | 2.61977 | 0.52083 | 1.57191 | 0.26712 | 0.15843 | 0.16502 | -1 |
| IL7r | 0.84983 | 0.17074 | 0.33957 | 0.05099 | 0.11689 | 0.09767 | -1 |
| Prex1 | 4.74359 | 2.11171 | 5.96069 | 0.89111 | 2.16427 | 3.19529 | -1 |
| Ripk3 | 5.57167 | 1.40391 | 3.08449 | 0.7314 | 1.69638 | 1.43489 | -1 |
| Tnfrsf21 | 5.73983 | 2.6164 | 5.60632 | 0.87021 | 3.15222 | 2.4239 | -1 |
| Map3k14 | 7.43416 | 3.72191 | 6.18767 | 0.80921 | 1.44801 | 0.37275 | -1 |
| Mapk13 | 1.50435 | 0.2996 | 1.70191 | 0.11185 | 0.58374 | 0.54965 | -1 |
| Mapk7 | 8.19793 | 5.6789 | 6.81499 | 0.54301 | 0.77457 | 0.10666 | -1 |
| Mapk8 | 17.115 | 14.9971 | 15.1203 | 1.78313 | 2.25425 | 1.17829 | -1 |
| Nhej1 | 1.53044 | 0.34043 | 1.8959 | 4.63948 | 3.44295 | 22.4196 | 1 |
| Prkdc | 0.48246 | 0.42849 | 1.27164 | 8.15381 | 5.66237 | 6.54881 | 1 |
| Lig4 | 0.85386 | 0.74801 | 1.05183 | 9.72393 | 6.49239 | 8.84368 | 1 |
| Rad50 | 3.04737 | 0.92399 | 2.37532 | 9.69359 | 21.1806 | 18.501 | 1 |
| Fen1 | 0.28101 | 0.15559 | 0.32605 | 5.03646 | 3.54004 | 4.12194 | 1 |
| **Gene symbols** | **Nod2^f/f^ 1** | **Nod2^f/f^ 2** | **Nod2^f/f^ 3** | *Nod2^△hep^* 1 | *Nod2^△hep^* 2 | *Nod2^△hep^* 3 | Upregulated (1)；Downregulated (-1) |
| Dntt | 0.94683 | 0.23558 | 0.34478 | 4.07536 | 3.14803 | 3.20507 | 1 |
| Xrcc4 | 0.21276 | 0.18919 | 0.64329 | 3.07996 | 2.42487 | 3.04944 | 1 |
| 1810064F22Rik | 0.24665 | 0.03263 | 0.50167 | 0.29004 | 3.67335 | 1.79857 | 1 |
| 2810007J24Rik | 40.6569 | 2.8965 | 13.6864 | 22.455 | 151.245 | 326.998 | 1 |
| Car3 | 167.754 | 6.25403 | 13.7554 | 60.4783 | 352.691 | 876.215 | 1 |
| Clec2h | 1.25508 | 0.15905 | 0.2678 | 0.86104 | 9.17269 | 3.11047 | 1 |
| Cyp7b1 | 4.00248 | 1.92642 | 7.00308 | 7.81498 | 50.1602 | 128.805 | 1 |
| Gm4956 | 0.79874 | 0.56449 | 7.75193 | 6.23898 | 22.4828 | 16.9858 | 1 |
| Hapln4 | 0.30769 | 0.15608 | 0.38449 | 1.57206 | 4.29482 | 3.2944 | 1 |
| Keg1 | 16.6686 | 4.84703 | 8.27676 | 24.6323 | 86.7316 | 125.173 | 1 |
| Lama3 | 0.05385 | 0.01489 | 0.08104 | 0.78285 | 0.2468 | 1.42062 | 1 |
| Serpine2 | 1.38274 | 0.82508 | 0.75291 | 6.2248 | 5.95332 | 16.571 | 1 |
| Slc15a5 | 0.07991 | 0.01944 | 0.50292 | 0.47098 | 2.95646 | 1.63593 | 1 |
| Mup10 | 348.336 | 89.0213 | 554.767 | 678.58 | 5701.54 | 4639.65 | 1 |
| Mup11 | 211.206 | 51.7678 | 391.996 | 513.53 | 4386.71 | 4037.12 | 1 |
| Mup14 | 101.749 | 26.2035 | 692.631 | 1417.08 | 11694.1 | 8449.65 | 1 |
| Mup18 | 354.386 | 87.551 | 546.394 | 663.268 | 5404.08 | 4807.54 | 1 |
| Mup20 | 312.382 | 81.465 | 531.038 | 2771.65 | 10261.1 | 12419.2 | 1 |
| Mup3 | 300.574 | 184.84 | 693.064 | 2771.65 | 9858.39 | 12419.2 | 1 |
| Mup8 | 38.9311 | 11.7564 | 108.482 | 170.4 | 1517.05 | 1091.93 | 1 |
| Mup9 | 411.997 | 92.8895 | 491.238 | 1008.37 | 10323.2 | 7494.18 | 1 |
| Nat8 | 0.75815 | 0.36279 | 2.29004 | 4.08255 | 26.325 | 31.3787 | 1 |
| Ttbk1 | 0.04456 | 0.00205 | 0.01633 | 0.0634 | 0.08735 | 0.13615 | 1 |
| Ttc25 | 0.04955 | 0.00681 | 0.09464 | 0.14409 | 0.80077 | 0.12824 | 1 |
| Ugt2b1 | 76.7665 | 30.3568 | 49.2575 | 97.4178 | 1515.71 | 1122.44 | 1 |
| Pik3r5 | 2.18824 | 0.79774 | 2.13582 | 0.1949 | 0.74312 | 0.27659 | -1 |
| Prkcq | 1.5861 | 1.03392 | 1.76972 | 0.2833 | 0.50035 | 0.45875 | -1 |
| Ptpn7 | 1.63877 | 0.9321 | 1.48235 | 0.10753 | 0.26304 | 0.41884 | -1 |
| Mmp9 | 4.66887 | 0.51613 | 1.85126 | 0.09287 | 1.00542 | 0.64237 | -1 |

**Online Table 6.**Summary of mass spectrometry (MS) analysis of NOD2 interactors.

| **Identified proteins** | **Unique Peptides** | **Protein**  **Accession** | **Protein Description** |
| --- | --- | --- | --- |
| CAVIN1 | 12 | Q6NZI2 | Caveolae-associated protein 1 |
| IMPDH2 | 11 | P12268 | Inosine-5'-monophosphate dehydrogenase 2 |
| TUBA1C | 10 | Q9BQE3 | Tubulin alpha-1C chain |
| MYH9 | 8 | P35579 | Myosin-9 |
| COR1C | 7 | Q9ULV4 | Coronin-1C |
| ACTB | 7 | P60709 | Actin, cytoplasmic 1 |
| ATPA | 6 | P25705 | ATP synthase subunit alpha, mitochondrial |
| LAP2B | 6 | P42167 | Lamina-associated polypeptide 2, isoforms beta/gamma |
| LRRF2 | 5 | Q9Y608 | Leucine-rich repeat flightless-interacting protein 2 |
| MYO1C | 5 | O00159 | Unconventional myosin-Ic |
| G6PD | 4 | P11413 | Glucose-6-phosphate 1-dehydrogenase |
| **LMNA** | **4** | **P02545** | **Lamin-A/C** |
| RUVB1 | 4 | Q9Y265 | RuvB-like 1 |
| LYN | 3 | P07948 | Tyrosine-protein kinase Lyn |
| CLH1 | 2 | Q00610 | Clathrin heavy chain 1 |
| SERA | 2 | O43175 | D-3-phosphoglycerate dehydrogenase |
| COPG1 | 2 | Q9Y678 | Coatomer subunit gamma |
| HSPA8 | 2 | E9PKE3 | Heat shock cognate 71 kDa protein |
| AP2M1 | 2 | Q96CW1 | AP-2 complex subunit mu |
| LACTB | 1 | P83111 | Serine beta-lactamase-like protein LACTB, mitochondrial |
| MYO6 | 1 | Q9UM54 | Unconventional myosin-VI |
| AL1A1 | 1 | P00352 | Retinal dehydrogenase 1 |
| GSHR | 1 | P00390 | Glutathione reductase, mitochondrial |
| SAM50 | 1 | Q9Y512 | Sorting and assembly machinery component 50 homolog |
| HNRH1 | 1 | P31943 | Heterogeneous nuclear ribonucleoprotein H |
| FXR1 | 1 | P51114 | Fragile X mental retardation syndrome-related protein 1 |
| AL3A1 | 1 | P30838 | Aldehyde dehydrogenase, dimeric NADP-preferring |
| TRIPB | 1 | Q15643 | Thyroid receptor-interacting protein 11 |

Bold value indicates Lamin-A/C.
