# Supplementary material for: Hepatic NOD2 promotes hepatocarcinogenesis via a RIP2-mediated proinflammatory response and a novel nuclear autophagy-mediated DNA damage mechanism
Source: J Hematol Oncol. 2021 Jan 7;14:9. doi: 10.1186/s13045-020-01028-4 (PMC7791875; doi:10.1186/s13045-020-01028-4)
Supplement: Supplementary file 2 — Additional file 2. Supplementary Materials and Methods. [file 13045_2020_1028_MOESM2_ESM.docx]

**Supplementary Materials and Methods**

**Histological analyses**

Fresh liver tissues were fixed in 4% paraformaldehyde and embedded in paraffin, then cut into 5 μm sections and stained with hematoxylin and eosin (H&E). For immunohistochemistry, sections were incubated with primary antibodies against NOD2, p-RIP2, ki67, F4/80, γ-H2AX or 8-OHdG overnight at 4°C, and secondary antibodies for 1 hour at 37°C if necessary. Samples were then developed with DAB and counterstained with hematoxylin. Primary antibodies and dilutions used were listed in **Additional file 1: Online Table 1**. Terminal deoxynucleotidyl transferase mediated dUTP nick end labeling (TUNEL) staining was performed using an *in situ* cell apoptosis detection kit (Roche, Indianapolis, IN, USA) to detect apoptosis of mouse liver cells. For Masson’s trichrome staining and Sirius red staining, sections were subjected to xylene and ethanol rehydration before staining. The staining was expressed as percent of the total area. Images were captured by microscope and analyzed using Image J software (NIH).

**Isolation and culture of primary mouse hepatocytes**

Primary hepatocytes were isolated from male mice (C57BL/6, 6-8 weeks) as described previously [1]. Briefly, i*n situ* liver perfusion was performed through portal vein with HBSS buffer, followed by collagenase IV (Sigma-Aldrich, St Louis, MO) solution for 30 min. Liver tissues were placed in a sterile cell culture dish containing collagenase IV, dissected and passed through cell strainers (70 μm). After centrifugation at 50 × g for 5 min, cell pellets were re-suspended in Dulbecco’s modified Eagle’s medium (DMEM). Hepatocytes were further purified by two additional cycles of centrifugation at 50 × g for 5 min and re-suspended in DMEM plus 10% fetal bovine serum (FBS). The hepatocyte viability was assessed by trypan blue staining. Because NOD2 is an intracellular protein, lipofectamine 300 was utilized to allow MDP to internalize into hepatocytes [2]. Primary hepatocytes were treated with MDP and Lipofectamine 3000 To activate the NOD2 receptor and control hepatocytes were treated with Lipofectamine 3000 alone.

**Cell transfection and infection**

Lentivirus was constructed as previously described [3,4]. Briefly, HA-tagged *NOD2*, *LaminA/C* cDNA or GFP-LC3 was cloned into the empty lentivector pCDH-CMV-MCS-EF1-GreenPuro (SBI), and the recombinant plasmid were co-transfected with psPAX2 and pMD2.G into 293T cells using Lipofectamine 3000 (Invitrogen, USA) according to the manufacturer's instructions. The lentivirus was collected at 72 h after transfection and was spun at 4°C, 3000 rpm for 10 min. The supernatant was collected and polybrene was added to the final concentration 8 g/ml. The mixture (2 ml) was added to the primary hepatocyte culture in a 60 mm dish with 3 ml of medium. The transduced cells were harvested after 48 h post-infection for further experiments. For plasmid transfection, the plasmids encoding GFP-tagged NOD2 or GFP-NOD2△NLS were transfected into indicated cells using Lipofectamine3000 and confirmed for the efficiency by western blotting analysis.

The primers for plasmids construction are shown in **Additional file 1: Online Table 2**.

**Reactive oxygen species (ROS) detection**

To detect ROS production in liver tissue, frozen liver sections were incubated with dihydroethidium (DHE, sigma) for 30 min in the dark and images were captured using fluorescent microscopy (Olympus, Shinjuku, Tokyo, Japan) were evaluated by image J.

**ALT and AST analyses**

Blood samples were collected from anesthetic mice and centrifuged at 3000 rpm for 5 min to collect serum. Serum levels of aspartate transaminase (AST) and alanine transaminase (ALT) were measured by using ALT assay and AST assay kits (Biovision, Milpitas, CA, USA) following the manufacturer’s procedures.

**Quantitative polymerase chain reaction (qPCR)**

Total RNA was isolated using TRIzol (Takara, Tokyo, Japan) and reverse transcribed into cDNA using PrimeScript RT Master Mix (Takara, Tokyo, Japan) on the ProFlex PCR system (Applied Biosystems, CA, USA). qPCR was performed with SYBR Green PCR master mix (Takara, Tokyo, Japan) using real-time PCR system (Applied Biosystems 7500, CA, USA). Expression data were normalized to GAPDH. Primer sequences were listed in **Additional file 1: Online Table 2**.

**Enzyme-linked immunosorbent assay (ELISA)**

Livers were dissected from each animal, and 50 mg of liver tissues were homogenized in 1 ml of Hanks' Balanced Salt Solution (HBSS)/10% FBS containing protease inhibitor and then centrifuged at 12, 000 × g for 10 min at 4 °C. Cytokines (IL-6, TNF-α, IL-1β and IFN-γ) concentration in the supernatant was determined with ELISA kits (Cat # 431304, Cat # 430916, Cat # 432616 and Cat # 430816; BioLegend Inc., San Diego, CA, USA) according to the manufacturer’s protocols. Tissue values were normalized to tissue wet weight and expressed as pg/mg of livers.

**Western blot analysis**

Liver tissues and cell samples were lysed in a RIPA buffer (Beyotime, Shanghai, China) with phenylmethylsulfonyl fluoride (PMSF; Beyotime, Shanghai, China) and a protease inhibitor cocktail (Roche, Indianapolis, IN, USA). Equal amounts of proteins were separated by SDS-PAGE and transferred to polyvinylidene fluoride (PVDF) membranes (Millipore, MA, USA). The membranes were blocked with 5% non-fat milk and incubated with primary antibodies overnight at 4°C. The membranes were then incubated with secondary antibodies at room temperature for 1 h and bands were developed using chemiluminescence substrate (Share-Bio, Shanghai, China) and the images were visualized using Tanon 4600 instrument (Tanon, Shanghai, China). Antibodies information was showed in **Additional file 1: Online Table 1**. Quantity One 4.6.2 software (Bio-Rad, Hercules, CA, USA) was used to measure the density of the protein bands.

**Taqman Copy Number Analysis**

Genomic DNA was isolated from 2 mm sections of murine or human FFPE slides by scrapings and tissue digested with Proteinase K overnight. After Proteinase K inactivation for 10 min at 95°C, DNA concentration was determined spectroscopically using a Nanodrop (Thermo Scientific) and appropriate genomic DNA was directly used for PCR reactions in duplicates. Genome instability analysis was carried out with TaqMan Copy Number Assays Kit (Cat# 4400291, Thermo Scientific, US) and *Polr2a* as internal reference according to the manufacturer’s instruction. *Fhit*, *Fgfr1* and *Fgr* were selected as genes of interest to tested genetic instability in human and mice as previously described [5]. Data analysis was performed using Copy Caller Software (Life Technologies, US).

**MDP Concentration measurement**

HCC specimens and normal liver tissues were collected from patients receiving surgical resection or liver transplantation at Zhongshan Hospital, Fudan University (n = 10), respectively. The MDP concentration of HCC and normal liver tissues was measured as previous described [6]. Briefly, one milliliter of HCC or normal liver sample was extracted with 1 mL chloroform/methanol/0.1 N HCl, 1:2:0.8 vol/vol/vol, and centrifuged at 8000 × g for 20 min. The aqueous phase was extracted with chloroform and 0.1 N HCl and recentrifuged. The subsequent aqueous phase was evaporated under vacuum, dissolved in H2O, and analyzed by high-performance liquid chromatography (Waters, USA). A reverse-phase Li-Chrosphere column (RPI 18, 250 nm × 4.6 mm, 5 μ) was used after guard cartridge (7.5 mm × 4.6 mm), using a solvent of acetonitrile:water 95:5 at a flow rate of 1 mL/min. Samples also were spotted on silica gel thin-layer chromatography plates and developed using a solvent (methylene chloride/methanol/water/acetic acid, 60:40:10:5, vol/vol/vol/vol) and then were detected using iodine vapor. Bands co-migrating with standard MDP on thin-layer chromatography were eluted from the silica gel and were treated with 0.025 mol/L periodic acid for 30 minutes at 37°C. Excess periodate was decreased by adding 2% wt/vol Na arsenite and then 0.1 mol/L 2-thiobarbituric acid was added in a boiling bath and reactions were developed by transferring into acidic butanol and absorbance was measured at 549 nm. A calibration curve was prepared by using 10-40 μg of sialic acid (Nacetylmuramic acid).

**Co-immunoprecipitation (co-IP) and** **mass spectrometry (MS) analysis**

Whole-cell extracts of clinical HCC tissues were prepared in lysis buffer (50 mM Tris-HCl, pH 7.5, 150 mM NaCl, 5 mM EDTA, 5 mM EGTA, 15 mM MgCl_2_, 0.1% NP40, pH 8.0, protease inhibitors). NOD2 antibody or appropriate control IgG was added to the lysate and incubated for 2 h on a rocking platform at 4°C, followed by addition of protein A/G agarose beads (sc-2003, Santa Cruz). After incubation at 4°C overnight, samples were washed three times with lysis buffer, re-suspended in SDS sample buffer, and boiled for 5 min. Supernatants were fractionated by SDS-PAGE. For MS analysis, separated protein bands in SDS-PAGE gels were stained with Coomassie brilliant blue, with control IgG samples analyzed in parallel to determine non-specific binding proteins. The bands were extracted from the gel and subjected to LC-MS/MS sequencing. The data was analyzed as previously described [7]. In brief, proteins were digested in gel, extracted, mixed with matrix and then spotted on sample plate. The masses of peptides were identified using time-of-flying (TOF, ABI 4700 protein analyzer, ABI). Data of MS and MS/MS were searched against Swiss-Port database (Homo Sapiens). Proteins identified by at least two unique peptides were considered as high potential target to be NOD2 binding partner.

**Confocal microscopy, transmission electron microscope (TEM) and live-cell imaging**

Cells were seeded on glass coverslips pre-coated with poly-l-lysine, fixed with 4% paraformaldehyde, permeabilized with 0.2% Triton X-100, and blocked in 10% BSA in PBST for 30 min at room temperature. Primary antibody incubation was carried out at 4°C overnight, followed by staining with secondary antibodies. DAPI (Beyotime, Shanghai, China) was used to locate cell nuclei. Images were captured on a Leica confocal microscope (Leica TCS SP8, Germany).

For double-labeled immunofluorescence of γ-H2AX and 53BP1, primary mice hepatocytes were pre-treated with DEN (100 μg/ml) for 1 h followed by simulating with or without MDP (10 μg/ml) for the indicated time points. Cells were then fixed and stained with γ-H2AX and 53BP1 antibodies and γ-H2AX and 53BP1 foci were imaged by confocal image.

For rescue experiments, primary mice hepatocytes were infected with negative control (control) and lamin A/C-overexpression (lamin A/C OE) viruses, respectively. Cells were then pre-treated with DEN (100 μg/ml) for 1 h followed by simulating with or without MDP (10 μg/ml) for 2 h.

For Lyso-Tracker Red staining, hepatocytes were treated with MDP (10 μg/ml) for 1 h, and then stained with 40nM Lyso-Tracker Red for 10min at 37°C. Cells were fixed with 4% paraformaldehyde and stained with primary antibody against lamin A/C, and then imaged by super-resolution microscopy (N-SIM, Nikon, Tokyo, Japan).

For TEM, cells were treated with or without MDP (10 μg/ml) for 2 h, and fixed with Glutaraldehyde solution followed by standard TEM procedures [8]. The images were then captured using a transmission electron microscope (HITACHI, Tokyo, Japan).

For live-cell imaging, adenovirus encoding mCherry-GFP-lamin A/C were infected into hepatocytes. mCherry-GFP-lamin A/C expressing cells were seeded onto a 35 mm glass bottom dish (NEST, 801001) and then treated with MDP (10 μg/ml). Time-lapse images were collected using Leica AF6000 every 15 min.

**GST pull-down assay**

GST, GST-lamin A/C, GST-NOD2 (1-618 aa) and GST-NOD2 (619-1040 aa) constructs were obtained from Hanyinbt (Shanghai, China) and verified by DNA sequencing. GST-tagged constructs were transformed into BL21(CD3) plyss competent cells (*Escherichia coli*, TIANGEN, Shanghai, China) and induced with isopropylthiogalactopyranoside (IPTG, 1mM) at 16°C for 14-16 h. Cell pellets were re-suspended in lysis buffer (PBS buffer with 1 mM PMSF, 1 mM DTT and 0.2 mg/ml lysozme) and disrupted by ultrasonic cell disruptor (200-300 w) and the suspensions were centrifuged at 10,000 × g for 10 min at 4°C.

For GST pull-down, the GST-fusion and GST protein were purified with glutathione agarose beads (Sigma- Aldrich) in PBS buffer at 4°C overnight and washed three times with PBS buffer containing 1% Triton-X and three times with PBS buffer. The beads were then incubated with the *in vitro* translated Lamin A/C or NOD2 at 4°C overnight. The beads were washed five times with PBS buffer containing 1% Triton-X and boiled with SDS sample buffer for 5 min for western blot analysis.

**Subcellular fractionation**

Cells were resuspended in harvest buffer (10 mM Hepes pH 7.9, 50 mM NaCl, 0.5 M sucrose, 0.1 mM EDTA, 0.5% Triton-100, phosphatase inhibitors and PMSF) and incubated on ice for 10 min. Nuclei were obtained by centrifugation at 12,000 rpm for 10 min at 4°C. Nuclei were washed three times with harvest buffer and dissolved in 1× loading buffer. The supernatant containing the cytosolic extract was also collected and dissolved in 5× loading buffer. For chromatin purification, the nuclear fractions were incubated in 1 × micrococcal nuclease reaction buffer (50 Mm Tris-HCl (PH 7.9, 25°C), 5 Mm CaCl_2_) containing 100 μg/ml BSA at 37°C for 30 min. The supernatant containing chromatin binding protein was obtained by centrifugation at 12,000 rpm for 10 min at 4°C. The cytoplasmic, nuclear and chromatin fractions were analyzed by SDS-PAGE. The validity of fractionation was detected by western blots using LaminB as the nuclear protein control, β-Tubulin as the cytosolic protein control and Histone 3 as the chromatin protein control.

**Comet assay**

The alkaline comet assay was performed following the manufacturer’s procedures (KeyGen, Nanjing, China). In brief, cells were treated DEN (100 μg/mL) for 1 h followed by stimulating with MDP (10 μg/ml) for 2 h. Cells were then harvested and resuspended in PBS (1 × 10^6^/ml). Microscope slides were first covered with a 0.5% normal melting point agarose. Subsequently, 1× 10^4^ cells were mixed with 75 μl of 0.7% low-melting point agarose at 37°C and layered onto the first agarose layer. Then, the slides were finally covered with a 0.7% low-melting point agarose. The slides were submerged in precooled lysis buffer for 2 h at 4°C and then incubated in alkaline electrophoresis buffer (1 mmol/l EDTA, 300 mmol/l NaOH) at room temperature for 1 h. After electrophoresis at 0.8 V/cm for 30 min, slides were neutralized using neutralization buffer (0.4 mM Tris-HCl, pH 7.5) and stained with propidium iodide. Images were captured on a fluorescence. The level of DNA damage was analyzed using comet tail moment (TM) and Olive tail moment (OTM) by CASP software (www.casplab.com). TM and OTM were scored for 50 cells/slides and three independent experiments were performed.

**Non-homologous end joining (NHEJ) assay**

The NHEJ assay was performed using the EJ5-GFP reporter assay as previously described [9]. Primary hepatocytes from control and *Nod2^△hep^* mice were plated in 6-well plates at 70% confluence and transfected with the EJ5-GFP reported plasmid [10] (Addgene plasmid # 44026, a gift from Jeremy Stark) using Lipofectamine 3000. For lamin A/C overexpression, plasmid encoding *LMNA* was transfected into hepatocytes from control mice together with the EJ5-GFP reported plasmid. On the following day, cells were transfected with the I-SceI plasmid [11] (Addgene plasmid # 26477, a gift from Maria Jasin) in the presence or absence of MDP (10 μg/ml) using Lipofectamine 3000 to induce double strand break production. After 48 hours, cells were washed with PBS, trypsinized, resuspended in hanks buffer and then the percentage of GFP positive cells was determined by flow cytometer (Becton Dickinson, Franklin Lakes, NJ, USA).

**Sanger sequencing on mutated genes in HCC tumor tissues**

Sanger sequencing on mutated genes in HCC tumor tissues Sequencing primers were designed to detect LMNA and TP53 mutations in HCC tumor tissues and were listed in **Additional file 1: Online Table 2**. We amplified genomic intervals containing the mutations by PCR. The PCR products were purified using AxyPrep DNA Gel Extraction Kit (Axygen) and sequenced using BigDye Terminator v3.1 Cycle Sequencing Kit (Life Technologies). Sequencing results were analyzed with ABI 3730xl DNA Analyzers.

**References**

1. Zhang X, Fan L, Wu J, Xu H, Leung WY, Fu K, et al. Macrophage p38alpha promotes nutritional steatohepatitis through M1 polarization. Journal of Hepatology. 2019;71:163-174.

2. Oh HM, Lee HJ, Seo GS, Choi EY, Kweon SH, Chun CH, et al. Induction and localization of NOD2 protein in human endothelial cells. Cellular Immunology. 2005;237:37-44.

3. Dong P, Wang X, Liu L, Tang W, Ma L, Zeng W, et al. Dampened VEPH1 activates mTORC1 signaling by weakening the TSC1/TSC2 association in hepatocellular carcinoma. Journal of Hepatology. 2020;S0168-8278(20)30400-1. doi:10.1016/j.jhep.2020.06.027

4. Liu LZ, Zhang Z, Zheng BH, Shi Y, Duan M, Ma LJ, et al. CCL15 Recruits Suppressive Monocytes to Facilitate Immune Escape and Disease Progression in Hepatocellular Carcinoma. Hepatology. 2019;69:143-159.

5. Boege Y, Malehmir M, Healy ME, Bettermann K, Lorentzen A, Vucur M, et al. A Dual Role of Caspase-8 in Triggering and Sensing Proliferation-Associated DNA Damage, a Key Determinant of Liver Cancer Development. Cancer Cell. 2017;32:342-359.e10.

6. Vavricka SR, Musch MW, Chang JE, Nakagawa Y, Phanvijhitsiri K, Waypa TS, et al. hPepT1 transports muramyl dipeptide, activating NF-kappaB and stimulating IL-8 secretion in human colonic Caco2/bbe cells. Gastroenterology. 2004;127:1401-9.

7. Bienvenu F, Jirawatnotai S, Elias JE, Meyer CA, Mizeracka K, Marson A, et al. Transcriptional role of cyclin D1 in development revealed by a genetic-proteomic screen. Nature. 2010;463:374-8.

8. Zhao G, Zhang P, Gong J, Zhang X, Wang P, Yin M, et al. Tmbim1 is a multivesicular body regulator that protects against non-alcoholic fatty liver disease in mice and monkeys by targeting the lysosomal degradation of Tlr4. Nature Medicine. 2017;23:742-752.

9. Benitez A, Liu W, Palovcak A, Wang G, Moon J, An K, et al. FANCA Promotes DNA Double-Strand Break Repair by Catalyzing Single-Strand Annealing and Strand Exchange. Molecular Cell. 2018;71:621-628.e4.

10. Bennardo N, Cheng A, Huang N, Stark JM. Alternative-NHEJ is a mechanistically distinct pathway of mammalian chromosome break repair. Plos Genetics. 2008;4:e1000110.

11. Richardson C, Moynahan ME, Jasin M. Double-strand break repair by interchromosomal recombination: suppression of chromosomal translocations. Genes & Development. 1998;12:3831-3842.
