# Supplementary material for: Hepatic NOD2 promotes hepatocarcinogenesis via a RIP2-mediated proinflammatory response and a novel nuclear autophagy-mediated DNA damage mechanism
Source: J Hematol Oncol. 2021 Jan 7;14:9. doi: 10.1186/s13045-020-01028-4 (PMC7791875; doi:10.1186/s13045-020-01028-4)
Supplement: Supplementary file 3 — Additional file 3: Online Fig. 1. NOD2 expression increases during the progression of DEN/CCl4- induced HCC, accompanied by an increase of p-RIP2 expression. Online Fig. 2. Receiver operating characteristic (ROC) curve analysis of NOD2 immunohistochemistry staining. Online Fig. 3. Generation of hepatocyte-specific Nod2-knockout mice (Nod2△hep). Online Fig. 4. Gene set enrichment analysis (GSEA) of the correlation between NOD2 expression and inflammation, as well as DNA damage in patients with HCC. Online Fig. 5. Hepatic NOD2 deficiency reduces DEN/CCl4-induced liver inflammatory response. Online Fig. 6. Increased MDP concentration in HCC tissues. Online Fig. 7. NOD2 agonist MDP promotes hepatocarcinogenesis. Online Fig. 8. NOD2 activation increases DEN/CCl4-induced inflammatory response in primary mouse hepatocytes. Online Fig. 9. NOD2 activation increases the activation of DNA damage response (DDR) pathway, and the level of γ-H2AX, in DEN-treated mouse hepatocytes, via an RIP2-independent manner. Online Fig. 10. The role of NOD2/lamin A/C interaction network in DNA damage repair process. Online Fig. 11. NOD2 harbors a nuclear localization sequence (NLS). Online Fig. 12. NOD2 activation regulates the protein stability of lamin A/C, but not the transcription of lamin A/C, through nuclear autophagy. Online. Fig. 13. NOD2 interacts with ATG16L1, a component of the LC3-conjugation complex. Online Fig. 14. Live-cell imaging analysis of mCherry-GFP-lamin A/C in hepatocytes. Online Fig. 15. Lamin A/C overexpression rescues the aggravating effect of NOD2 activation on DEN-induced DNA damage in hepatocytes. Online Fig. 16. Lamin A/C expression decreases during the progression of DEN/CCl4-induced HCC. Online Fig. 17. MDP increases DEN/CCl4-induced inflammatory response in Lamn△hep, but not in Rip2/Lamn△hep mice. Online Fig. 18. LMNA mutation associated with increased genomic instability in HCC. [file 13045_2020_1028_MOESM3_ESM.pdf]

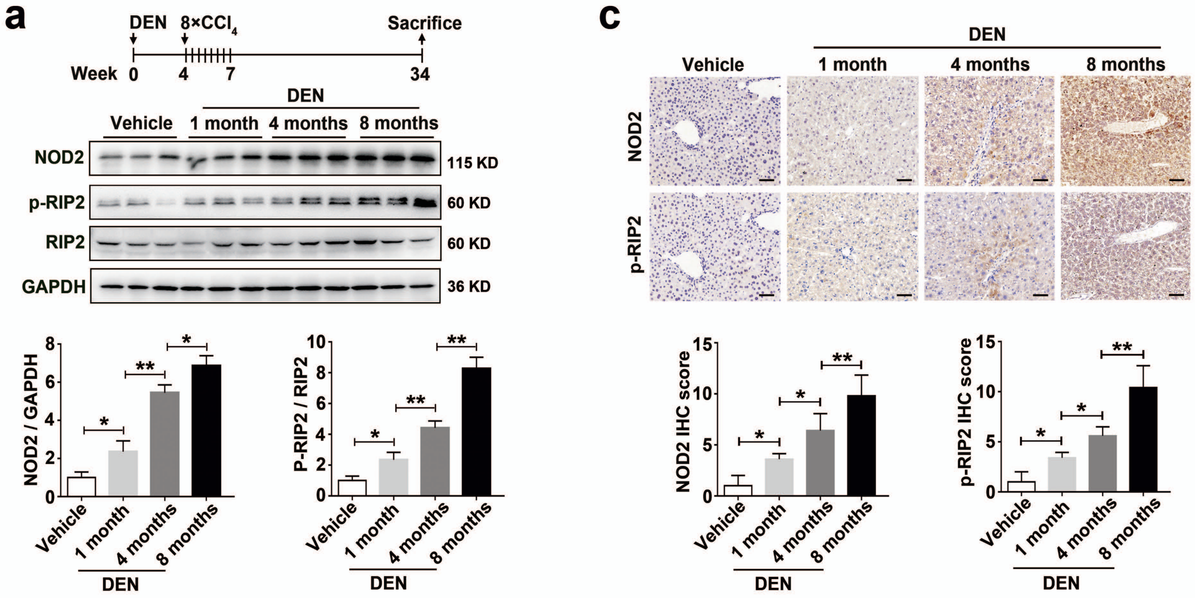

a
DEN
8×CCl4
Sacrifice
Week 0 4 7 34
DEN
Vehicle
1 month
4 months
8 months
NOD2
115 KD
p-RIP2
60 KD
RIP2
60 KD
GAPDH
36 KD
NOD2 / GAPDH
P-RIP2 / RIP2
c
NOD2
p-RIP2
NOD2 IHC score
p-RIP2 IHC score

Online Fig. 1

Online Fig. 1. NOD2 expression increases during the progression of DEN/CCl_4-induced HCC, accompanied by an increase of p-RIP2 expression.

a Representative expression patterns of liver NOD2 and p-RIP2 and quantification of relative intensity in different phases (1, 4, and 8 months) of DEN/CCl_4-induced HCC model in wild-type (WT) mice, n=3. **b** Representative immunohistochemistry images of liver NOD2 and p-RIP2 (top panel) and the quantification of immunohistochemistry scores (bottom panel) in different phases of DEN/CCl_4-induced HCC model in WT mice, n = 5. Data were shown as mean ± SD and significance was determined using ordinary one-way ANOVA with a Sidak test (**a** and **b**). $*P < 0.05$, $**P < 0.01$. Scale bar, 50 μm.

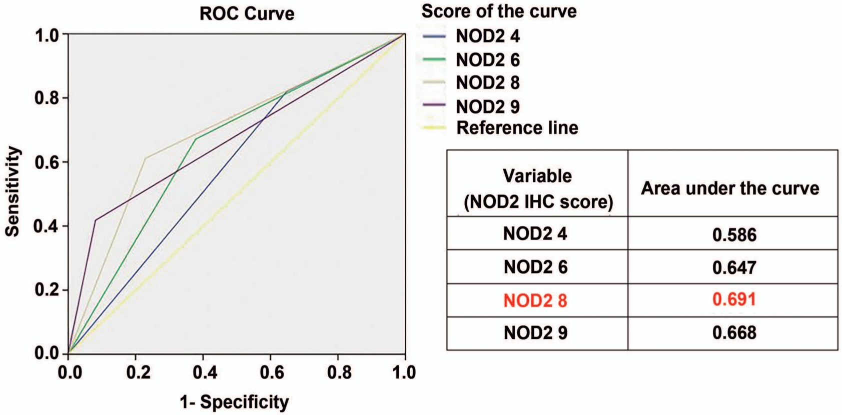

Variable (NOD2 IHC score)	Area under the curve
NOD2 4	0.586
NOD2 6	0.647
NOD2 8	0.691
NOD2 9	0.668

Online Fig. 2

Online Fig. 2. Receiver operating characteristic (ROC) curve analysis of NOD2 immunohistochemistry staining.

ROC curve analysis showed that the optimal cut-off value of NOD2 was 8 and the area under the curve (AUC) was 0.691 (95% CI, 0.602-0.780, $P < 0.001$). NOD2 $\geq$ 8 was considered as high expression of NOD2 and NOD2 < 8 was considered as low expression.

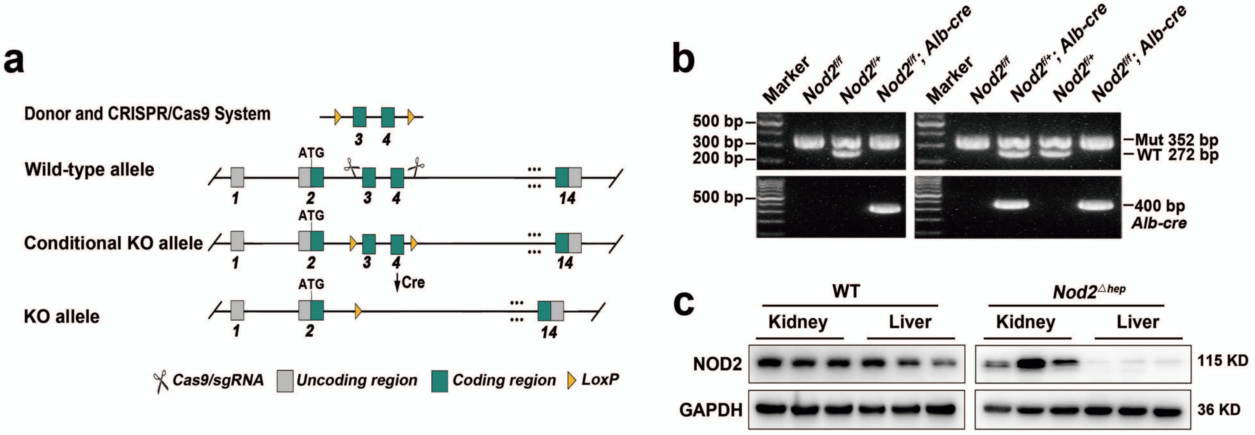
a
Donor and CRISPR/Cas9 System
3
4
Wild-type allele
ATG
1
2
3
4
14
Conditional KO allele
ATG
1
2
3
4
14
Cre
KO allele
ATG
1
2
14
Cas9/sgRNA
Uncoding region
Coding region
LoxP
b
Marker
Nod2f/f
Nod2f/+
Nod2f/f; Alb-cre
Marker
Nod2f/f
Nod2f/+; Alb-cre
Nod2f/+
Nod2f/f; Alb-cre
500 bp
300 bp
200 bp
Mut 352 bp
WT 272 bp
500 bp
400 bp
Alb-cre
c
WT
Nod2△hep
Kidney
Liver
Kidney
Liver
NOD2
GAPDH
115 KD
36 KD

Online Fig. 3

Online Fig. 3. Generation of hepatocyte-specific *Nod2*-knockout mice (*Nod2*$^{\triangle hep}$).

a Schematic representation of the strategy to generate the *Nod2* conditional allele. **b** Genotyping of *Nod2*$^{loxp/loxp}$ (*Nod2*$^{f/f}$), *Nod2*$^{loxp/+}$ (*Nod2*$^{f/+}$) and *Nod2*$^{loxp/loxp}$ Alb-cre (*Nod2*$^{\triangle hep}$). The wild-type (WT) allele yields an amplicon of 272 bp, while the floxed allele yields an amplicon of 352 bp. **c** Western blotting analysis of NOD2 protein in the livers and kidneys of WT and *Nod2*$^{\triangle hep}$ mice.

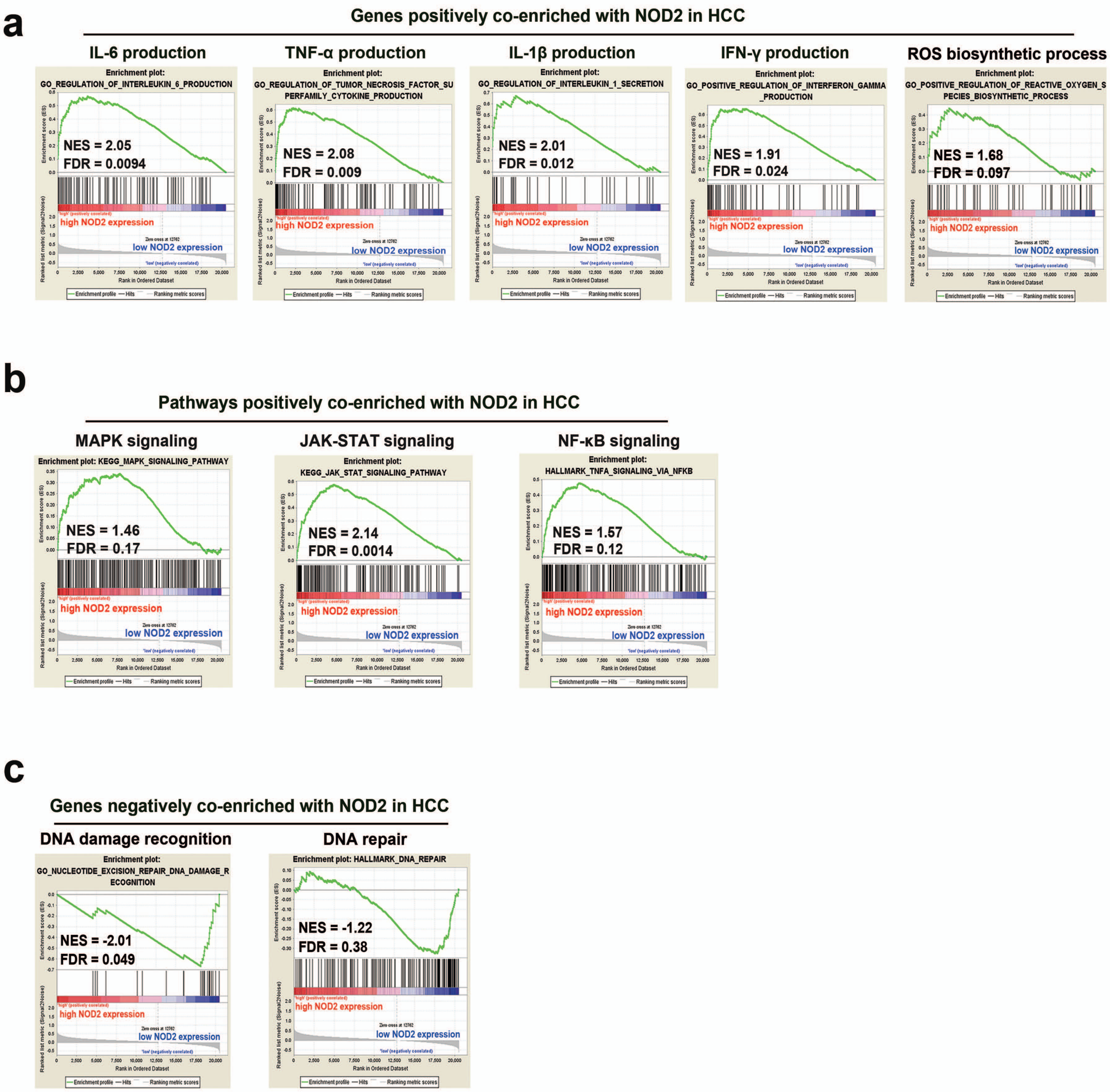
a
Genes positively co-enriched with NOD2 in HCC
IL-6 production
NES = 2.05
FDR = 0.0094
TNF-α production
NES = 2.08
FDR = 0.009
IL-1β production
NES = 2.01
FDR = 0.012
IFN-γ production
NES = 1.91
FDR = 0.024
ROS biosynthetic process
NES = 1.68
FDR = 0.097
b
Pathways positively co-enriched with NOD2 in HCC
MAPK signaling
NES = 1.46
FDR = 0.17
JAK-STAT signaling
NES = 2.14
FDR = 0.0014
NF-κB signaling
NES = 1.57
FDR = 0.12
c
Genes negatively co-enriched with NOD2 in HCC
DNA damage recognition
NES = -2.01
FDR = 0.049
DNA repair
NES = -1.22
FDR = 0.38
high NOD2 expression
low NOD2 expression

Online Fig. 4

Online Fig. 4. Gene set enrichment analysis (GSEA) of the correlation between NOD2 expression and inflammation, as well as DNA damage in patients with HCC. HCC cohort data were downloaded from the TCGA database. **a-b** GSEA reveals the positive correlation of NOD2 expression with inflammatory cytokines production (IL-6, TNFα, IL-1β, and IFN-γ), with ROS biosynthetic process (**a**), and with pro-inflammatory pathways (**b**) in patients with HCC. **c** GSEA reveals the negative correlation of NOD2 expression with DNA damage recognition, and with DNA repair in patients with HCC. NES, normalized enrichment score; FDR, false discovery rate.

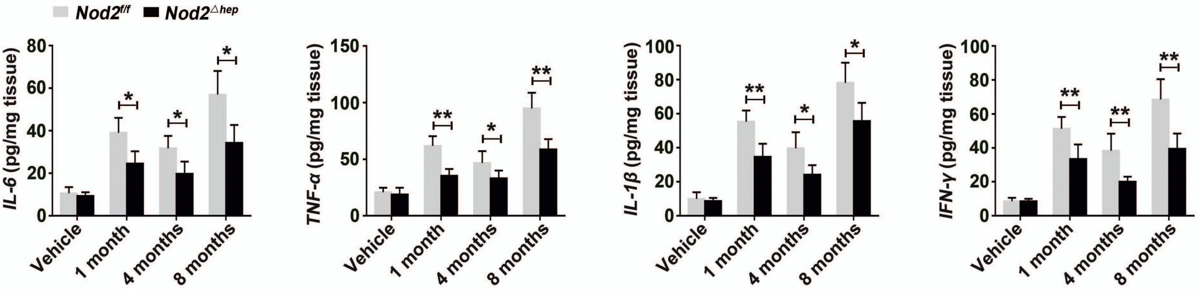

Nod2f/f
Nod2△hep
IL-6 (pg/mg tissue)
TNF-α (pg/mg tissue)
IL-1β (pg/mg tissue)
IFN-γ (pg/mg tissue)
Vehicle
1 month
4 months
8 months

Online Fig. 5

Online Fig. 5. Hepatic NOD2 deficiency reduces DEN/CCl_4-induced liver inflammatory response.

The concentration of IL-6, TNFα, IL-1β and IFN-γ in livers of *Nod2*$^{f/f}$ and *Nod2*$^{\triangle hep}$ mice treated with DEN/CCl_4 for the indicated time points was determined by ELISA, n = 6. Data were shown as mean ± SD and significance was determined using ordinary one-way ANOVA with a Sidak test. $^*P < 0.05$, $^{**}P < 0.01$.

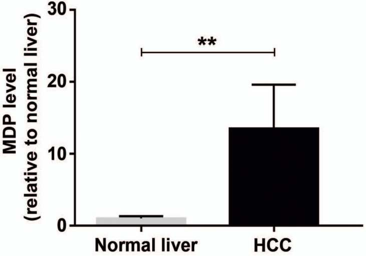
MDP level
(relative to normal liver)
30
20
10
0
**
Normal liver
HCC

Online Fig. 6

Online Fig. 6. Increased MDP concentration in HCC tissues.

MDP level was increased in HCC tissues (n = 10), compared to normal liver tissues (n = 10). Data were shown as mean ± SD and significance was determined using student's t test. ***P* < 0.01.

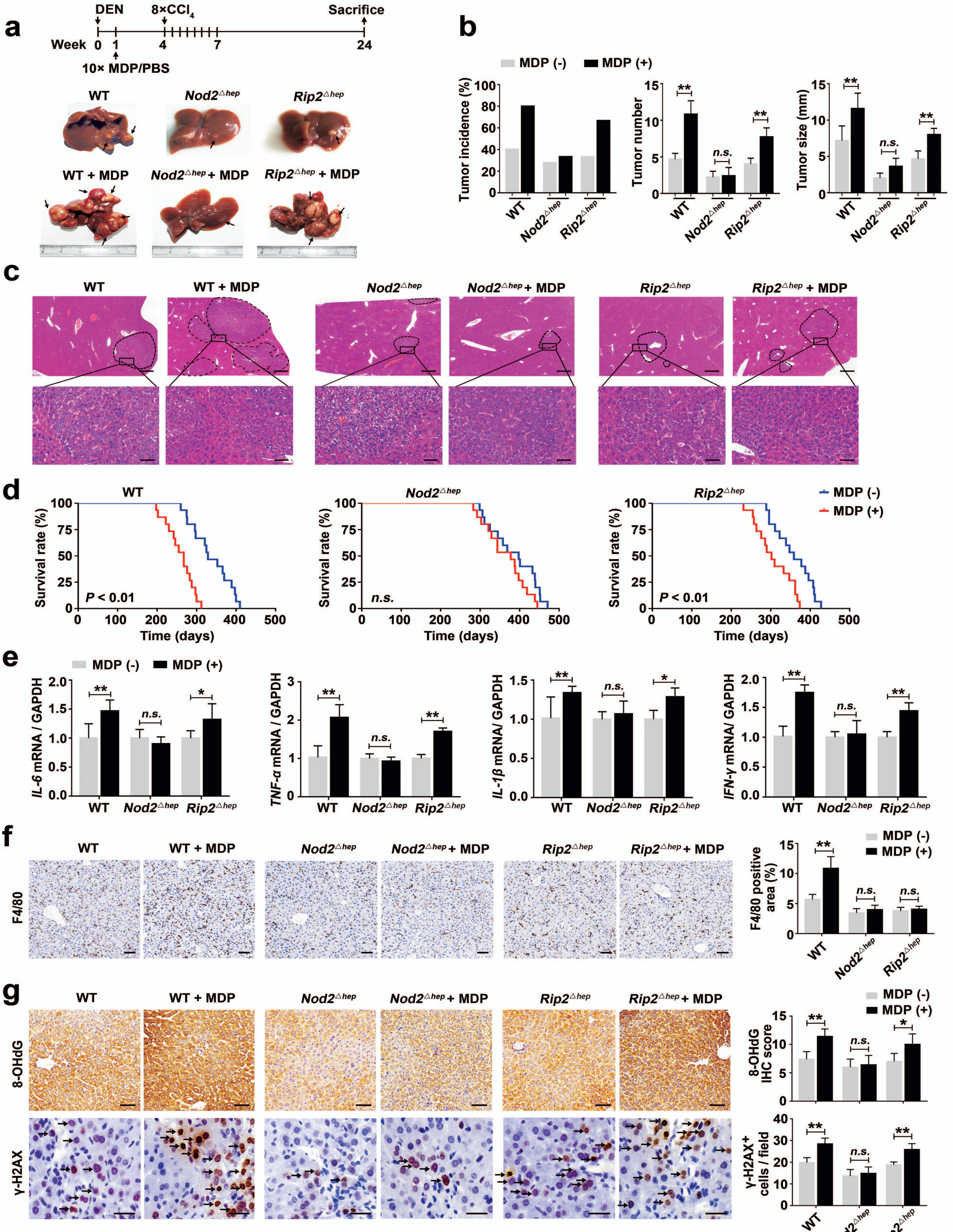
a
DEN
8×CCl4
Sacrifice
Week 0 1 4 7 24
10× MDP/PBS
WT
Nod2△hep
Rip2△hep
WT + MDP
Nod2△hep + MDP
Rip2△hep + MDP
b
MDP (-)
MDP (+)
Tumor incidence (%)
Tumor number
Tumor size (mm)
n.s.
c
d
Survival rate (%)
Time (days)
P < 0.01
n.s.
e
IL-6 mRNA / GAPDH
TNF-α mRNA / GAPDH
IL-1β mRNA/ GAPDH
IFN-γ mRNA/ GAPDH
f
F4/80
F4/80 positive area (%)
g
8-OHdG
γ-H2AX
8-OHdG IHC score
γ-H2AX+ cells / field

Online Fig. 7

Online Fig. 7. NOD2 agonist MDP promotes hepatocarcinogenesis.

a-b Male wild-type (WT), *Nod2$^{\Delta hep}$*, and *Rip2$^{\Delta hep}$* mice were injected with DEN (25 mg/kg, i.p.) at the age of 14-16 days followed by 8 injections of CCl_4 (1.2 ml/kg, i.p., biweekly, starting 4 weeks after DEN injection) and either MDP (100 µg/mice, every other day) or PBS via intraperitoneal injection for 10 times starting three week before the first injection of CCl_4. Mice were sacrificed 6 months after DEN injection. Representative gross appearance of the liver with tumors (**a**), as well as tumor incidence (**b**, left panel; untreated WT, n = 15; treated WT, n = 15; untreated *Nod2$^{\Delta hep}$*, n = 18; treated *Nod2$^{\Delta hep}$*, n = 15; untreated *Rip2$^{\Delta hep}$*, n = 15; treated *Rip2$^{\Delta hep}$*, n = 15) and quantification of tumor number (**b**, middle panel) and tumor size (**b**, right panel) in WT (untreated n = 6; treated n = 12), *Nod2$^{\Delta hep}$* (untreated n = 5; treated n = 5), and *Rip2$^{\Delta hep}$* (untreated n = 7; treated n = 10) mice. Arrowheads indicate tumors in liver. **c** Representative H&E staining images of liver sections from WT, *Nod2$^{\Delta hep}$*, and *Rip2$^{\Delta hep}$* mice at 6 months after DEN/CCl_4 treatment. Scale bar, 500 µm (top panel) or 50 µm (bottom panel). **d** Survival rate of WT, *Nod2$^{\Delta hep}$*, and *Rip2$^{\Delta hep}$* mice. n = 15. **e** Relative mRNA expression of *IL-6*, *TNF-α*, *IL-1β*, and *IFN-γ* in liver tissues of WT, *Nod2$^{\Delta hep}$*, and *Rip2$^{\Delta hep}$* mice at 1 month after DEN/CCl_4 treatment, n = 5. **f-g** Representative immunohistochemistry images and quantification of F4/80 (**f**), 8-OHdG and γ-H2AX (**g**) in livers of WT, *Nod2$^{\Delta hep}$*, and *Rip2$^{\Delta hep}$* mice at 1 month after DEN/CCl_4 treatment, n = 5. Arrowheads indicate γ-H2AX$^+$ cells. Scale bar, 50 µm (top panel) or 25 µm (bottom panel). Data were shown as mean ± SD and significance was determined using student's t test (**b**, **d**, **f**, and **g**). **P* < 0.05, ***P* < 0.01, *n.s.*, not significant.

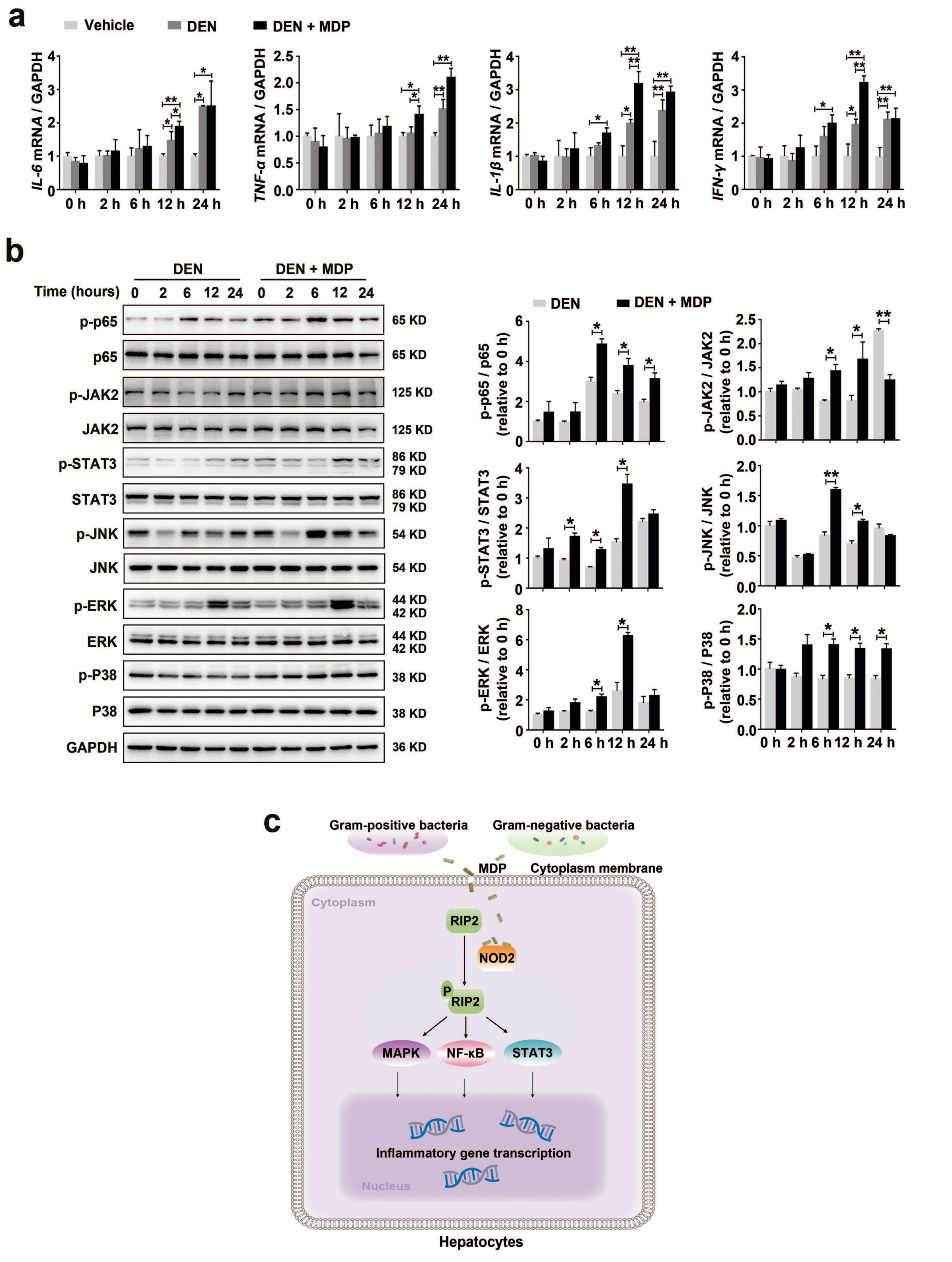

a
Vehicle
DEN
DEN + MDP
IL-6 mRNA / GAPDH
TNF-α mRNA / GAPDH
IL-1β mRNA / GAPDH
IFN-γ mRNA / GAPDH
0 h
2 h
6 h
12 h
24 h
b
DEN
DEN + MDP
Time (hours)
p-p65
p65
p-JAK2
JAK2
p-STAT3
STAT3
p-JNK
JNK
p-ERK
ERK
p-P38
P38
GAPDH
65 KD
125 KD
86 KD
79 KD
54 KD
44 KD
42 KD
38 KD
36 KD
p-p65 / p65 (relative to 0 h)
p-JAK2 / JAK2 (relative to 0 h)
p-STAT3 / STAT3 (relative to 0 h)
p-JNK / JNK (relative to 0 h)
p-ERK / ERK (relative to 0 h)
p-P38 / P38 (relative to 0 h)
c
Gram-positive bacteria
Gram-negative bacteria
MDP
Cytoplasm membrane
Cytoplasm
RIP2
NOD2
P
MAPK
NF-κB
STAT3
Inflammatory gene transcription
Nucleus
Hepatocytes

Online Fig. 8

Online Fig. 8. NOD2 activation increases DEN/CCl_4-induced inflammatory response in primary mouse hepatocytes.

a Primary hepatocytes from wild-type (WT) mice were treated with DEN (100 μg/ml) in the present or absent of MDP (10 μg/ml) for the indicated time points (0, 2, 6, 12 and 24 h), then the mRNA expression of *IL-6*, *TNF-α*, *IL-1β*, and *IFN-γ* was tested using qPCR, n = 3. **b** Primary hepatocytes were treated as described in (**a**), and then the activation of NF-κB, JAK-STAT3, and MAPK pathways was examined using western blotting. Densitometric analysis of band intensity was shown in bar graph, n = 3. **c** Proposed schematic of hepatic NOD2 regulatory mechanisms in the inflammatory response. Data were shown as mean ± SD and significance was determined using ordinary one-way ANOVA with a Sidak test (**a** and **b**). *$P < 0.05$, **$P < 0.01$.

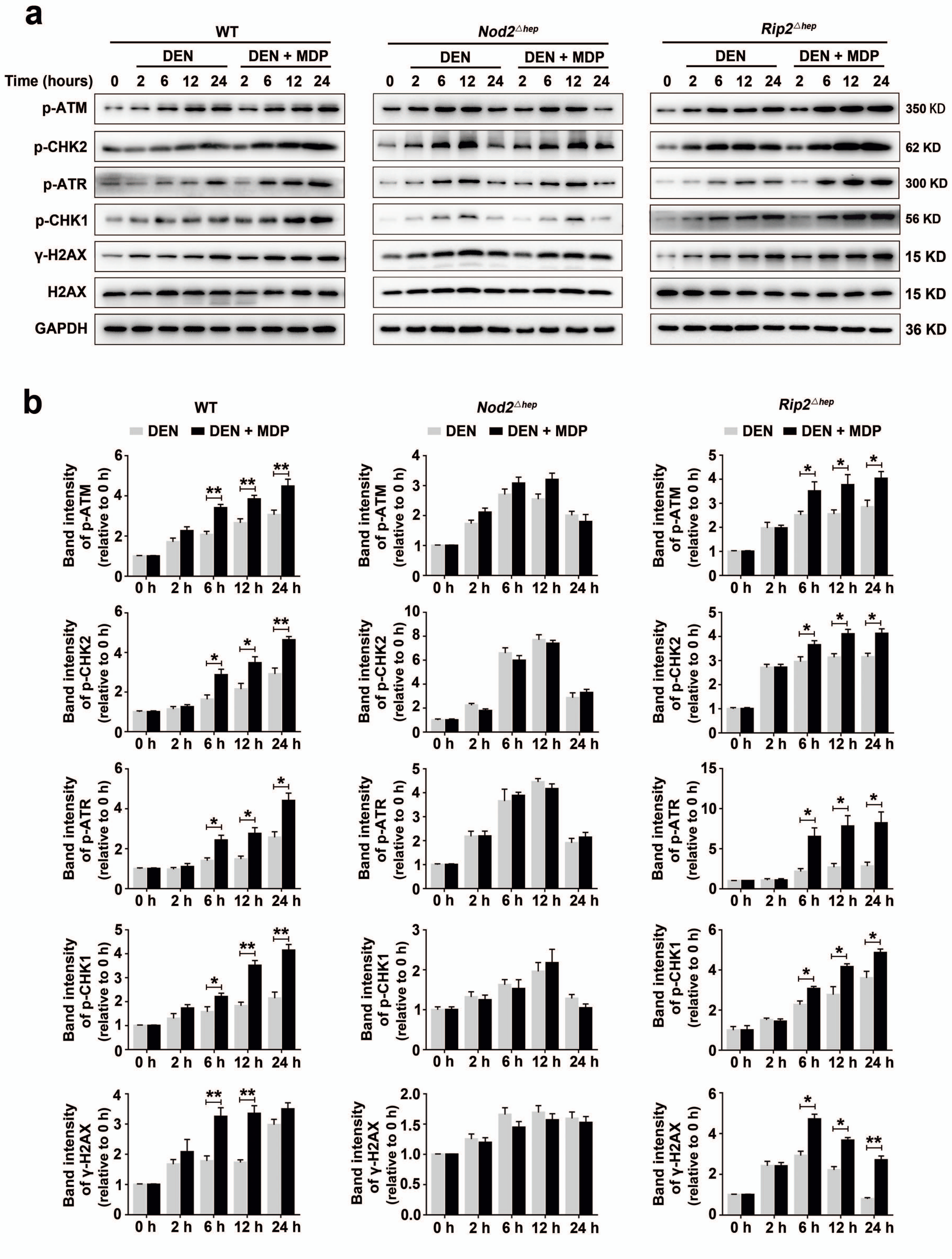
a
WT
Nod2△hep
Rip2△hep
DEN
DEN + MDP
Time (hours) 0 2 6 12 24 2 6 12 24
p-ATM
p-CHK2
p-ATR
p-CHK1
γ-H2AX
H2AX
GAPDH
350 KD
62 KD
300 KD
56 KD
15 KD
15 KD
36 KD
b
Band intensity of p-ATM (relative to 0 h)
Band intensity of p-CHK2 (relative to 0 h)
Band intensity of p-ATR (relative to 0 h)
Band intensity of p-CHK1 (relative to 0 h)
Band intensity of γ-H2AX (relative to 0 h)
0 h 2 h 6 h 12 h 24 h

Online Fig. 9

Online Fig. 9. NOD2 activation increases the activation of DNA damage response (DDR) pathway, and the level of γ-H2AX, in DEN-treated mouse hepatocytes, via an RIP2-independent manner.

a Primary hepatocytes from wild-type (WT), $Nod2^{\Delta hep}$, and $Rip2^{\Delta hep}$ mice were treated with DEN (100 μg/ml) in the present or absent of MDP (10 μg/ml)) for the indicated time points (0, 2, 6, 12, and 24 h). Then the activation of DDR pathway (ATM/CHK2, ATR/CHK1) and the level of γ-H2AX in cells were examined by western blot analysis. **b** Densitometric analysis of band intensity of western blots, n = 3. Data were shown as mean ± SD and significance was determined using student's t test (**b**). *$P < 0.05$, **$P < 0.01$.

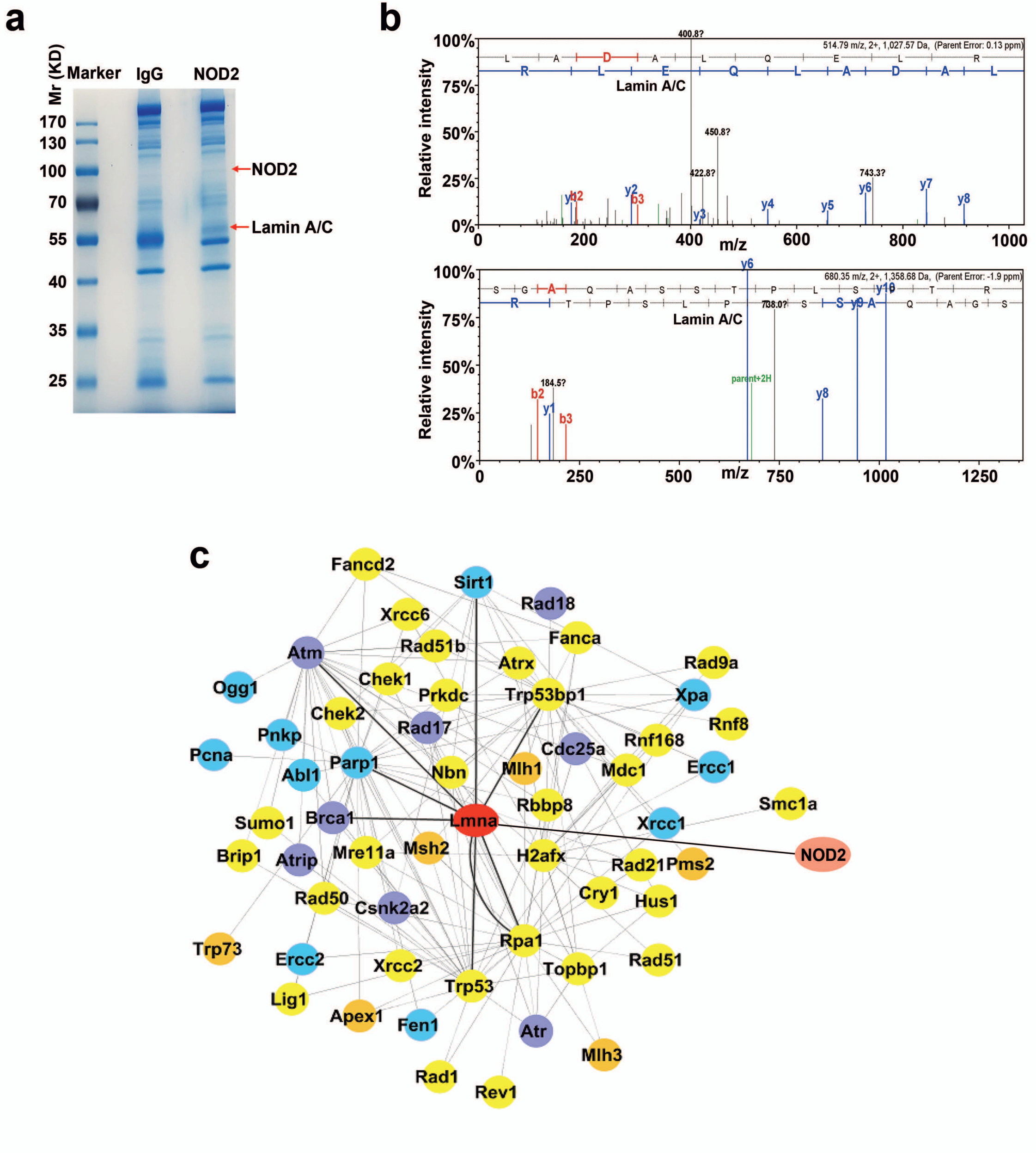

Online Fig. 10

Online Fig. 10. The role of NOD2/lamin A/C interaction network in DNA damage repair process.

a Coomassie brilliant blue-stained SDS-PAGE gel of NOD2 immunoprecipitates from HCC samples. IgG immunoprecipitates were loaded as control. **b** Representative mass spectrometry (MS) spectra of lamin A/C peptide. **c** Protein-protein interaction network of lamin A/C with proteins related to DNA damage repair. Yellow node: DNA double strand break repair signaling; purple node: DNA damage response signaling; blue node: excision repair signaling; orange node: mismatch repair signaling. RNA-seq data (*Nod2*$^{\triangle hep}$ and *Nod2*$^{f/f}$ transcriptomes), String database (https://string-db.org/) and Cytoscape 3.2.1 (http://www.cytoscape.org/) were used to output the network.

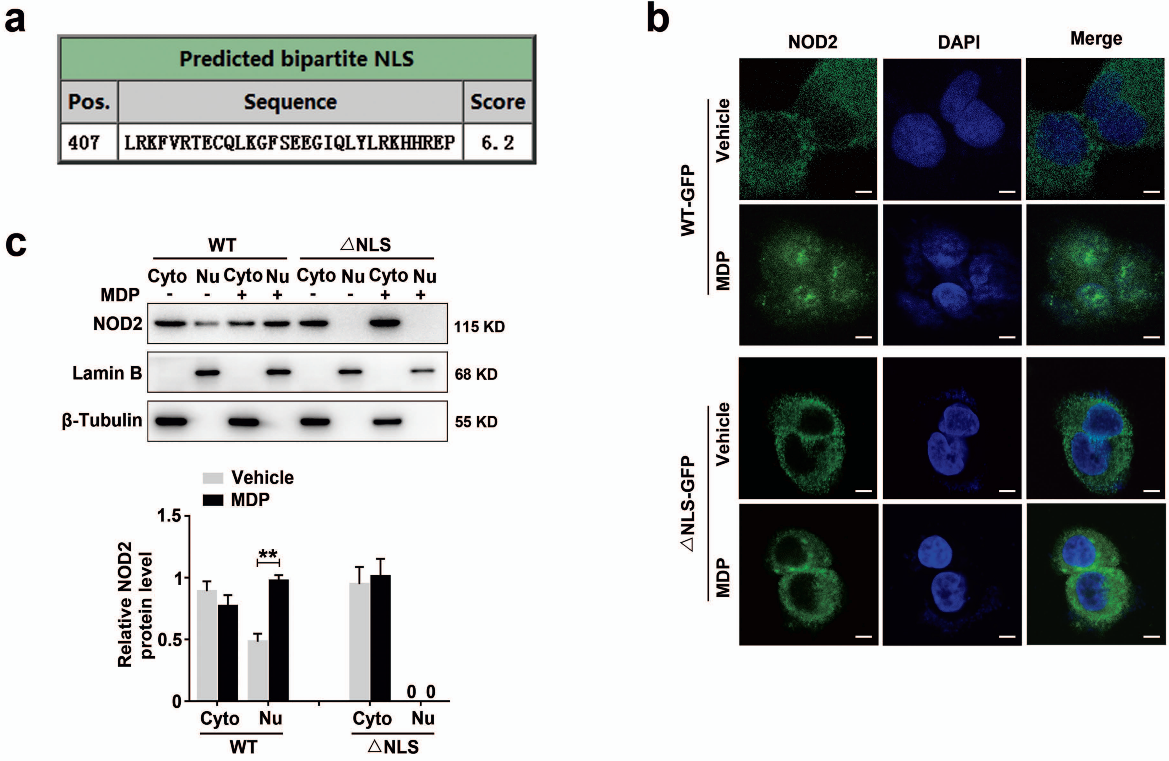

Online Fig. 11

Online Fig. 11. NOD2 harbors a nuclear localization sequence (NLS).

a Identification of NLS on NOD2 (amino acids 407-436) using cNLS mapper program (http://nls-mapper.iab.keio.ac.jp/cgi-bin/NLS_Mapper_form.cgi). **b** NLS motif (amino acids 407-436) is required for the nuclear translocation of NOD2. 293T cells were transfected with GFP-tagged full-length NOD2, or GFP-tagged NOD2 with NLS fragment amino acids 407-436 deletion (△NLS) plasmid. After MDP (10 μg/ml) treatment for 1 h, the protein levels of NOD2 in nucleus and cytoplasm were observed using confocal microscope. Scale bar, 10 μm. **c** 293T cells were treated with or without MDP (10 μg/ml) for 1 h, then nuclear and cytoplasmic subcellular fractions were extracted as described in Materials and method. The protein levels of NOD2 in nucleus and cytoplasm were examined using western blotting. Quantitative analysis of band intensity was shown as bar graph. Data were shown as mean ± SD and significance was determined using student's t test (**c**). n = 3; $^{**}P < 0.01$.

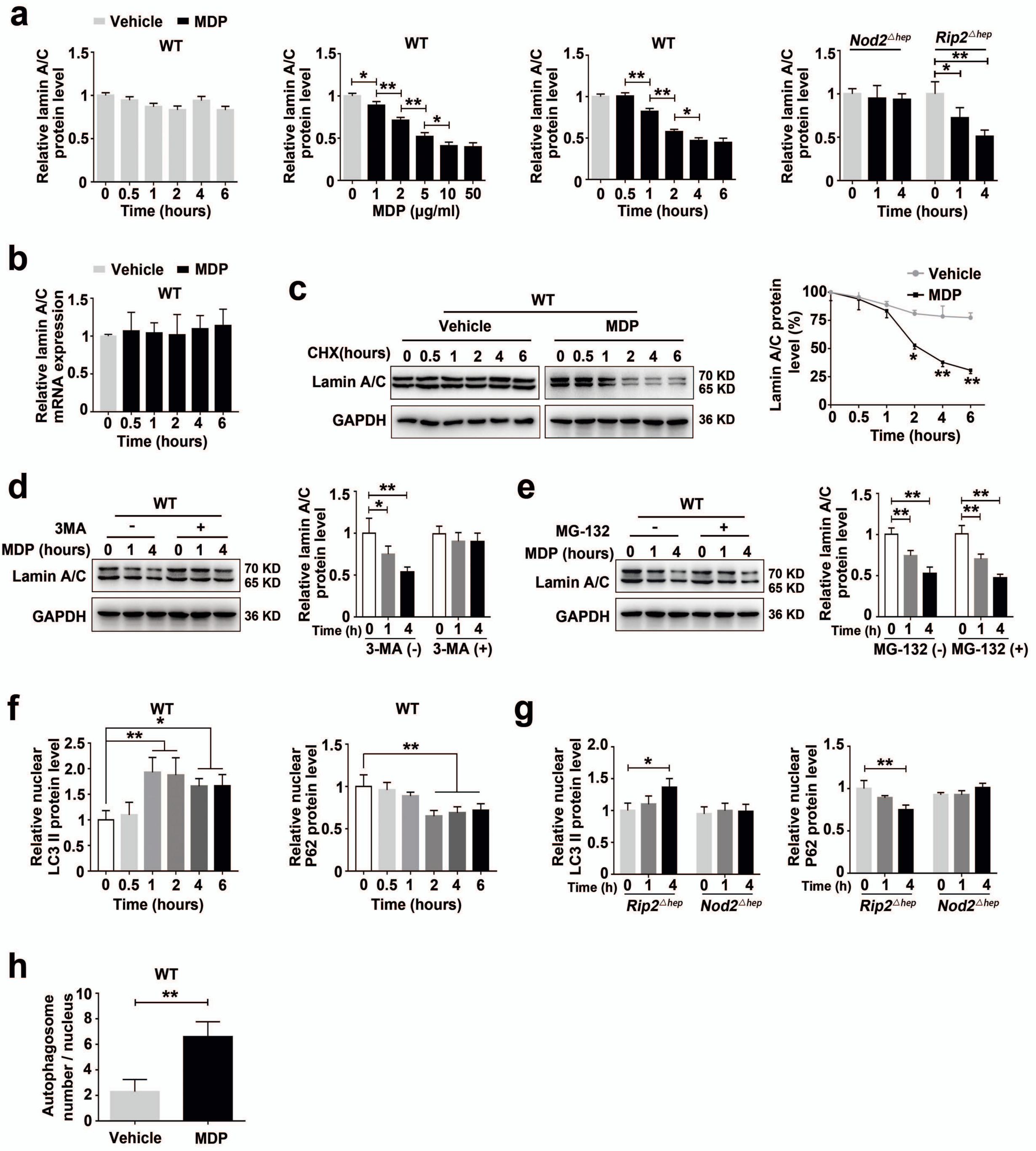

Online Fig. 12

Online Fig. 12. NOD2 activation regulates the protein stability of lamin A/C, but not the transcription of lamin A/C, through nuclear autophagy.

a Densitometric quantification of immunoblotting results corresponding to Figure **5d** showed that NOD2 activation reduces the protein level of lamin A/C in primary hepatocytes, n = 3. **b** NOD2 activation does not affect the mRNA expression of lamin A/C. Primary hepatocytes from wild-type (WT) were treated with MDP (10 μg/ml) for the indicated time points (0, 0.5, 1, 2, 4, and 6 h), then the mRNA expression of lamin A/C was tested by qPCR, n = 3. **c** NOD2 activation reduces the protein stability of lamin A/C. Left panel: primary hepatocytes were pretreated with cycloheximide (CHX, 100 μM) for 1 h followed by vehicle or MDP (10 μg/ml) stimulation, then the protein level of lamin A/C was examined at the indicated time using western blotting. Right panel: quantification of lamin A/C protein level, n = 3. **d-e** NOD2 activation induces the degradation of lamin A/C protein through autophagy pathway. Primary hepatocytes were pre-incubated with or without MG-132 (30 μM, **d**) or 3-MA (10 mM, **e**) for 1 h. Then cells were treated with MDP (10 μg/ml) for the indicated times and the lamin A/C protein level was determined by western blotting. Column bar graph showing densitometric quantification of lamin A/C band intensity relative to GAPDH, n = 3. **f** Densitometric quantification of immunoblotting results of nucleus LC3-II or P62 band intensity relative to lamin B in Figure **5e**, n = 3. (g) Densitometric quantification of immunoblotting results corresponding to Figure **5f**, n = 3. (h) Quantification of nuclear autophagosomes corresponding to Figure **5g** showing that NOD2 activation induces the formation of nuclear autophagosomes, n = 10. Data were shown as mean ± SD and

significance was determined using Ordinary two-way (**c**) or one-way ANOVA with a Sidak test (**a, d**, **e**, **f**, **g**), and student's t test (**h**). *$P < 0.05$, **$P < 0.01$.

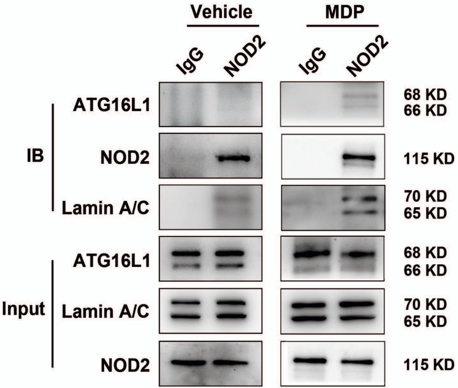

Online Fig. 13

Online. 13. NOD2 interacts with ATG16L1, a component of the LC3-conjugation complex.

Hepatocytes expressed HA-NOD2 were treated with or without MDP (10 μg/ml) for 1 h, then the interaction between NOD2 and ATG16L1 was determined by co-immunoprecipitation analysis.

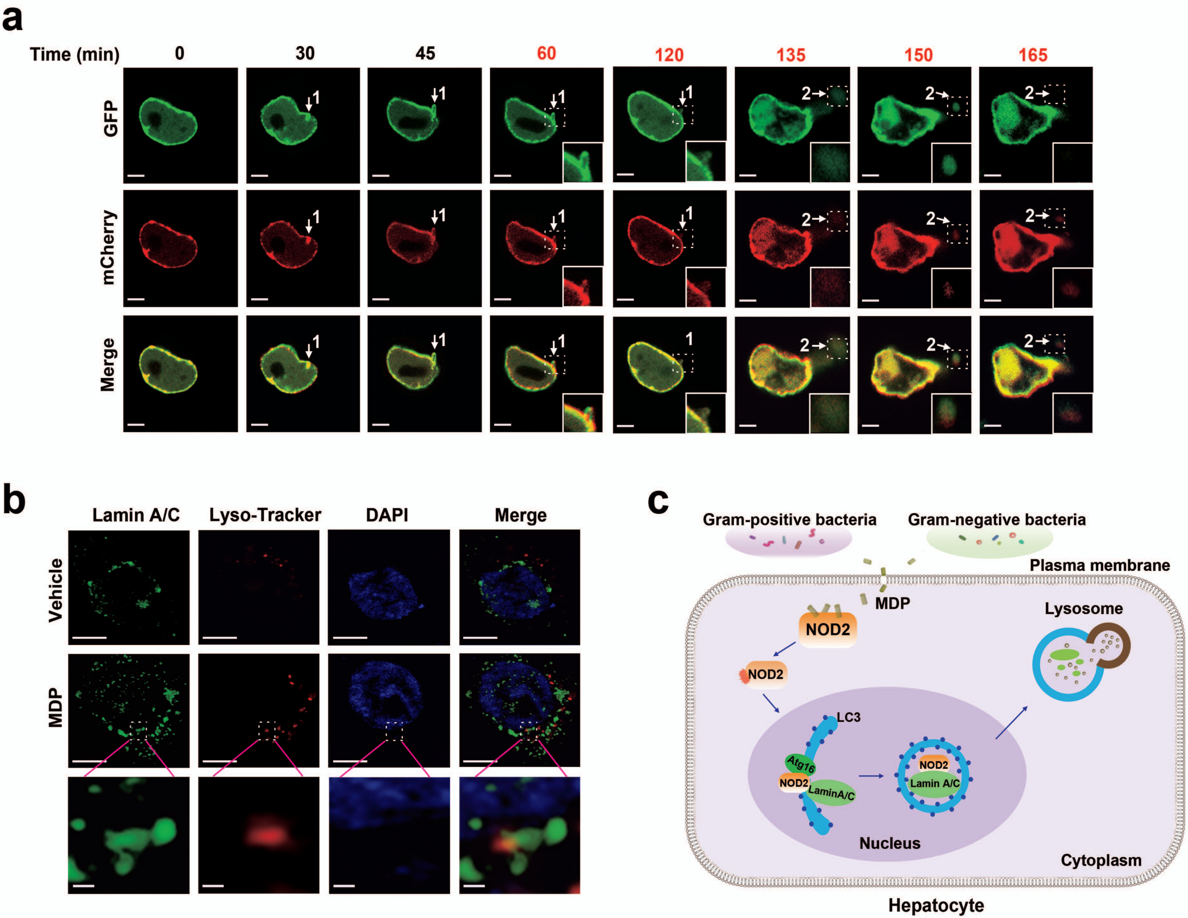

Online Fig. 14

Online Fig. 14. Live-cell imaging analysis of mCherry-GFP-lamin A/C in hepatocytes.

a Time-lapse image of mCherry–GFP–lamin A/C hepatocytes showed that lamin A/C translocates from nucleus to cytoplasm and enters lysosome for degradation after MDP treatment. Primary hepatocytes infected with adenovirus encoding mCherry-GFP-lamin A/C were treated with MDP (10 μg/ml) for the indicated time points. Nucleus-to-cytoplasm transport events were labeled sequentially as indicated. Arrows showing that nuclear membrane blebs in event 1 and lamin A/C enters the lysosome from the cytoplasm in event 2. Scale bar, 5 μm. **b** MDP-treated hepatocytes were stained with Lyso-Tracker Red and lamin A/C (green), and imaged by super-resolution microscopy. Scale bar, 5 μm (top and middle panel) or 50 μm (bottom panel). **c** Proposed schematic of hepatic NOD2-initiated nuclear autophagy.

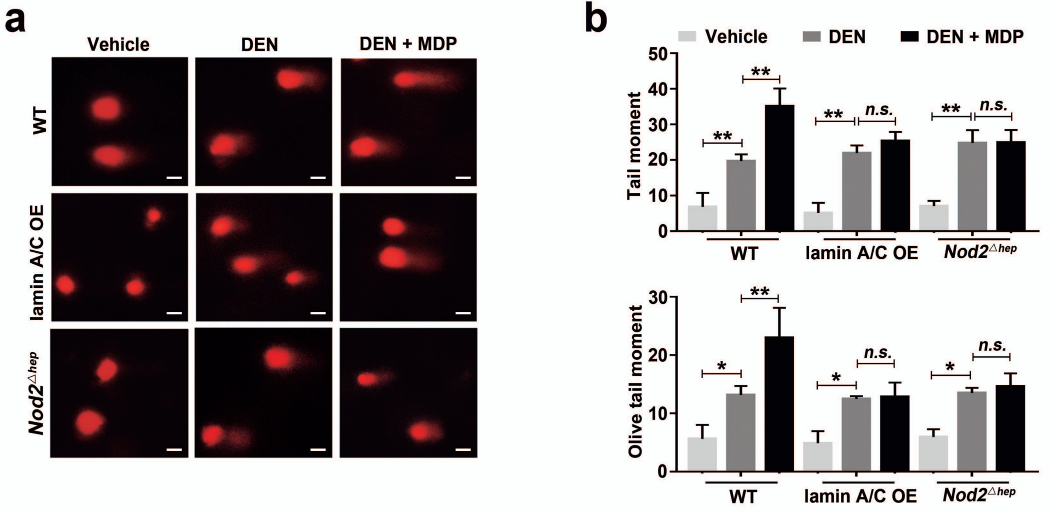

Online Fig. 15

Online Fig. 15. Lamin A/C overexpression rescues the aggravating effect of NOD2 activation on DEN-induced DNA damage in hepatocytes.

a The aggravating effect of NOD2 activation on DNA double-strand break (DSB) was largely rescued by overexpressing lamin A/C in DEN-treated hepatocytes. wild-type (WT) mice hepatocytes, WT mice hepatocytes transfected with lamin A/C-overexpression (lamin A/C OE) plasmids, and *Nod2*$^{\Delta hep}$ mice hepatocytes were treated with DEN (100 μg/ml) for 1 h, and the DSB was detected by Comet Assay. **b** Quantification of the amount of DNA damage, as measured by the comet tail moment and olive tail moment. At least 50 cells were scored. Data were shown as mean ± SD and significance was determined using Ordinary one-way ANOVA with a Sidak test (**b**). **P* < 0.05, ***P* < 0.01, *n.s*, not significant. Scale bar, 100 μm.

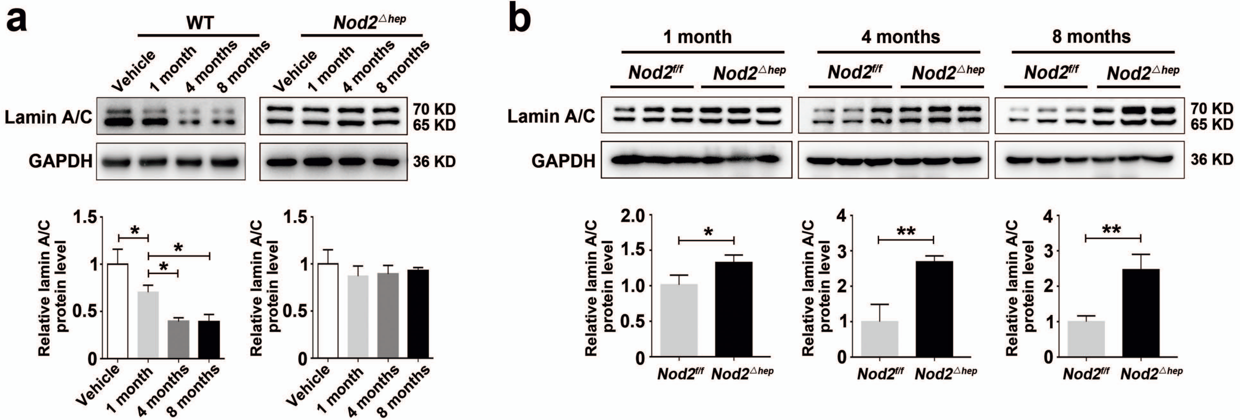

Online Fig. 16

Online Fig. 16. Lamin A/C expression decreases during the progression of DEN/CCl_4-induced HCC.

a Western blotting analysis and quantification of lamin A/C in the livers of WT and *Nod2*$^{\triangle hep}$ mice at the indicated time points (1, 4, and 8 months) after DEN/CCl_4 treatment, n = 3. **b** Western blotting analysis and quantification of lamin A/C in the livers of *Nod2*$^{f/f}$ and *Nod2*$^{\triangle hep}$ mice at the indicated time points after DEN/CCl_4 treatment, n = 3. Data were shown as mean ± SD and significance was determined using Ordinary one-way ANOVA with a Sidak test (**a**) and student's t test (**b**). $*P < 0.05$, $**P < 0.01$.

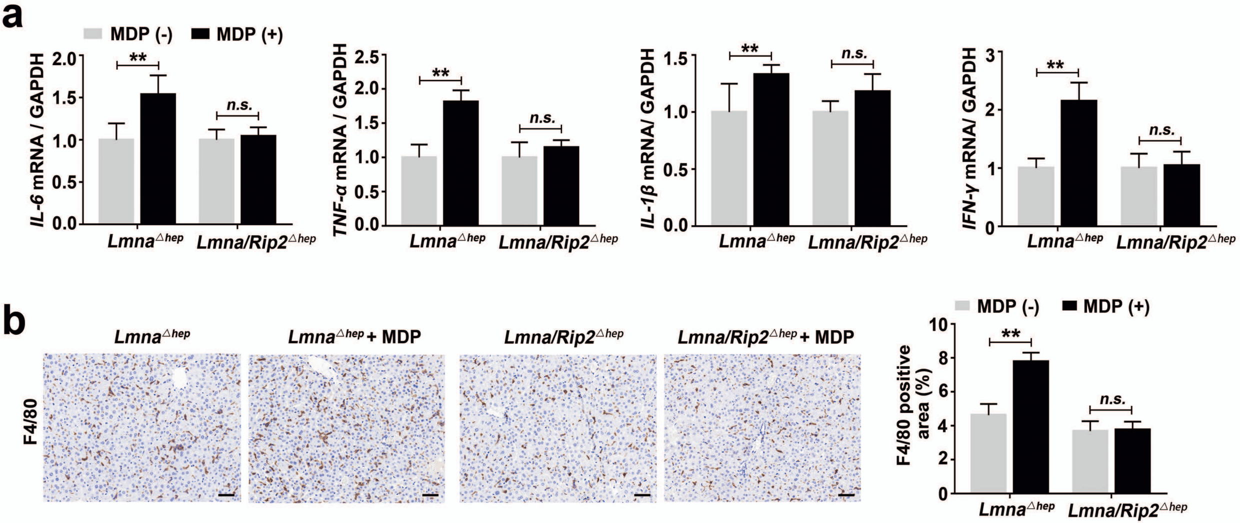

Online Fig. 17

Online Fig. 17. MDP increases DEN/CCl_4-induced inflammatory response in *Lamn*$^{\Delta hep}$, but not in *Rip2/Lamn*$^{\Delta hep}$ mice.

Male *Lamn*$^{\Delta hep}$ and *Rip2/Lamn*$^{\Delta hep}$ mice were treated as Figure **7a**. Mice were sacrificed 1 month after DEN injection. **a** Relative mRNA expression of *IL-6*, *TNF-α*, *IL-1β*, and *IFN-γ* in liver tissues of *Lamn*$^{\Delta hep}$ and *Rip2/Lamn*$^{\Delta hep}$ mice, n = 5. **b** Representative immunohistochemistry images and quantification of F4/80 in liver tissues of *Lamn*$^{\Delta hep}$ and *Rip2/Lamn*$^{\Delta hep}$ mice, n = 5. Scale bar, 50 μm. Data were shown as mean ± SD and significance was determined using ordinary two-way ANOVA with a Sidak test (**a** and **b**). $^{**}P < 0.01$, *n.s.*, not significant.

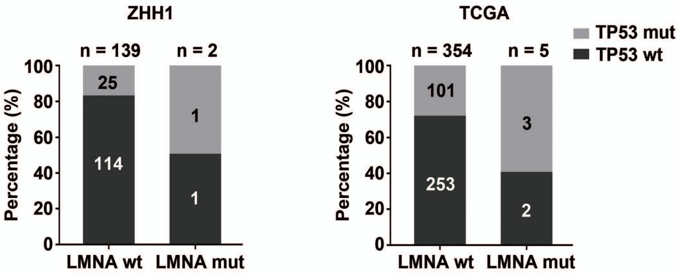

Online Fig. 18

Online Fig. 18. LMNA mutation associated with increased genomic instability in HCC.

Mutation status of LMNA and TP53 in ZHH1 cohort (n = 141) and in TCGA HCC cohort (n = 359).
